# Inheritance of gene expression throughout fruit development in chili pepper

**DOI:** 10.1038/s41598-021-02151-z

**Published:** 2021-11-22

**Authors:** Christian Escoto-Sandoval, Neftalí Ochoa-Alejo, Octavio Martínez

**Affiliations:** 1 Centro de Investigación y de Estudios Avanzados del Instituto Politécnico Nacional (Cinvestav), Unidad de Genómica Avanzada (Langebio), Irapuato Guanajuato, 36824 México; 2 Centro de Investigación y de Estudios Avanzados del Instituto Politécnico Nacional (Cinvestav), Departamento de Ingeniería Genética, Unidad Irapuato, Irapuato Guanajuato, 36824 México

**Keywords:** Computational biology and bioinformatics, Genetics, Molecular biology, Plant sciences

## Abstract

Gene expression is the primary molecular phenotype and can be estimated in specific organs or tissues at particular times. Here we analyzed genome-wide inheritance of gene expression in fruits of chili pepper (*Capsicum annuum* L.) in reciprocal crosses between a domesticated and a wild accession, estimating this parameter during fruit development. We defined a general hierarchical schema to classify gene expression inheritance which can be employed for any quantitative trait. We found that inheritance of gene expression is affected by both, the time of fruit development as well as the direction of the cross, and propose that such variations could be common in many developmental processes. We conclude that classification of inheritance patterns is important to have a better understanding of the mechanisms underlying gene expression regulation, and demonstrate that sets of genes with specific inheritance pattern at particular times of fruit development are enriched in different biological processes, molecular functions and cell components. All curated data and functions for analysis and visualization are publicly available as an R package.

## Introduction

Relative gene expression, as measured by genome wide methods such as RNA-Seq^1^ or microarrays, constitutes the primary molecular phenotype. The study of gene expression, or “*transcriptomics*”, favors the understanding of the molecular processes that give rise to the whole phenotype, and thus contributes to solve the central problem of how the interaction of genotype and environment produces the phenotype^2^.

There are reports of substantial heritability for gene expression in various organisms^3^; for example, a study of human cell lines^4^ found that 31% of the genes had significant heritability, while in *Arabidopsis* it has been reported a median heritability of 28.6% and 74.7% when calculating the parameter from parental data or a RIL population, respectively^5^.

Heterosis or hybrid vigor, the superior performance of the $F_1$ hybrid compared with their parents^6–10^, is an important phenomenon whose molecular foundations remain enigmatic. Nevertheless, heterosis cannot be directly inferred from gene expression, because while the former is directly measured in traits of interest, as for example yield, the latter rarely has a direct and unique effect in such traits. In fact, variations in the presence of genes, the presence of novel beneficial alleles and modified levels of gene expression in hybrids may all contribute to the heterotic phenotypes^11^; thus, heterosis for different characters is likely due to different sets of genes.

Hybrid vigor is classified as “mid-parent heterosis”, when the $F_1$ is superior than the mean of the two parents, or “better parent heterosis”, when the $F_1$ is superior to the best of the two parents^6,11^. There are different genetical models to explain heterosis, as dominance, overdominance, epistasis or pseudo-dominance^11–14^. However, not single model can always explain heterosis, and it must be studied on a case by case basis, evaluating the contribution of each possible model.

Recent studies are helping to unveil the molecular bases of heterosis. For example^11^, mentions studies that found between 5 and 10% of genes with differential expression in maize that have effects on heterosis, and also discuss evidence of allelic interactions that lead to novel hybrid expression patterns. In their review of the molecular basis of heterosis^6^, discuss how complex gene expression networks, detected in maize, rice and *Arabidopsis* for different developmental stages and tissues could contribute to improve our understanding of the molecular basis of heterosis, but also underline that no uniform global expression patterns were observed in these studies. Related to this point, a gene expression analysis in maize inbreds and hybrids with varying levels of heterosis, suggests that transcriptional diversity at specific sets of genes may influence heterosis for different traits^7^. In^12^, the authors remark that heterosis is an environmentally modified phenotype, and suggest that an integrated “*phenomics*” approach—including not only genomics and transcriptomics, but also QTL-based phenotyping followed by map-based cloning should be employed in order to understand the role of heterosis in evolution and the domestication of plants. In sunflower heterotic gene pools were developed through the use of crop-wild relatives^14^, and in that paper the authors suggest that there may be underexploited variation within open-pollinated varieties, and thus mining older lines for useful traits could be fruitful. Furthermore, in^15^ the authors showed the importance of variation and inheritance of small RNAs in maize inbreds and $F_1$ hybrids, while^16^ demonstrated that pathway expression complementation contributes to biomass heterosis in *Arabidopsis*, while^17^ brings new insights into the molecular mechanisms of heterosis during the cabbage head development.

In *Capsicum*, the study in^18^ reports that crosses performed with local chili pepper from Thailand showed significant heterosis for yield, and fruit size and quality in some of the $F_1$’s. Also in chili pepper^19^, found significant heterosis for yield components and fruit quality in the $F_1$ of various crosses between Indian varieties. Also in this crop^20^, addressed heterosis estimation in 72 hybrids, finding positive heterosis of more than 30% for yield related components in 8 of those hybrids. Even when there is currently no direct link between hybrid vigor and individual gene expression in *Capsicum*, and heterosis in this crop might be attributed to the complex molecular regulation that is required for the manifestation of even a single phenotypical trait^6^, we assert that the detailed knowledge of gene expression inheritance patterns within crosses will help in the understanding of this phenomenon.

In summary, gene expression is a heritable quantitative character that affects agronomical fitness and constitutes a molecular phenotype^21^. Genome wide estimation of gene expression using high-throughput methods, as RNA-Seq, will allows us to understand how the variation in this parameter arose and to predict how it is most likely to evolve in the future^22^.

On the other hand, gene expression studies constitute the base to identify gene regulatory networks^23^, and these in turn help to understand the dynamics of processes, as the *Arabidopsis* flower organ specification^24^, or the changes induced by osmotic stress in that plant^25^. Also, in^26^, we presented a robust network of cell cycle genes with a time shift in expression, which explains some of the differences between domesticated and wild phenotypes in chili pepper.

Gene expression can be characterized by ordering the values of that parameter in the parents and the $F_1$ of the cross, and then performing statistical tests to evaluate significance. In this way, we determine gene expression inheritance classes and models that can be used to examine this quantitative phenotype in a formal manner. Even when gene expression has been intensely studied in humans and model organisms to determine eQTL^3,5,27^, these studies generally omit the basic step of characterizing gene expression inheritance.

Here we studied gene expression by RNA-Seq in a cross between a domesticated and a wild accession of chili pepper in both directions (female $\times$ male and male $\times$ female) at seven time points during fruit development. At each one of the crosses and fruit development times we characterized gene expression inheritance, showing that this framework unveils interesting aspects of the transcriptome landscape. We also show that gene expression inheritance depends on the fruit development stage as well as on the direction of the cross, and that specific inheritance types are enriched in genes with different biological processes, molecular functions, and cell components.

## Materials and methods

### Plant accessions and fruit development sampling

We confirm that all plant materials used in this study comply with the relevant institutional, national, and international guidelines and legislation.

To study gene expression inheritance, we selected two accessions of chili pepper which presented broad differences in both, fruit phenotype and domesticated history. The first was the large fruit domesticated accession *Capsicum annuum* cv. CM334, also known as “Criollo de Morelos 334”^28^ and the second was the wild accession of *C. annuum* var. *glabriusculum*^29^ known as “Chiltepin” or “Piquín Querétaro”. Thereafter, these two accessions are labeled with keys “CM” (Criollo de Morelos 334, Parent 1; $P_1$) and “QU” (Piquín Querétaro, Parent 2; $P_2$) for the domesticated and wild accessions, respectively.

We performed the cross $P_1 \times P_2$ in both directions to obtain the corresponding $F_1$’s as shown in Table 1.

**Table 1:** Definitions of crosses as function of parents and $F_1$ keys.

Cross id	Definition	$P_1$	$P_2$	$F_1$
$\mathbf {C}_1$	CM female $\times$ QU male	CM	QU	CQ
$\mathbf {C}_2$	QU female $\times$ CM male	CM	QU	QC

In Table 1 we see that in the cross denoted as $\mathbf {C}_1$ pollen donator was the accession QU, resulting in the $F_1$ with key “CQ”, while in the cross $\mathbf {C}_2$ pollen donator was the accession CM, resulting in the $F_1$ with key “QC”.

To obtain robust estimates of relative gene expression during fruit development, we sampled total RNA from developing fruits of genotypes CM, QU, CQ and CQ (Table 1) at 0, 10, 20, 30, 40, 50 and 60 Days After Anthesis (DAA). Two RNA-Seq libraries (biological replicates) were constructed, sequenced and mapped to the *Capsicum* reference genome for each one of the $4 \times 7 = 28$ combinations of genotype $\times$ time of fruit development, for a total of $28 \times 2 = 56$ RNA-Seq libraries. The total number of clean reads mapped to the reference genome from these 56 libraries was of 985.8 million with an average of approximately 17.6 million per library. This study was performed in parallel with the one reported by us in^26^, and full details of plant cultivation, fruit sampling, RNA-Seq libraries construction, sequencing and curation can be consulted in the supplementary material of that article. The full RNA-Seq data have been deposited in NCBI’s Gene Expression Omnibus^30^ and are accessible through the GEO Series accession number GSE165448.

### Gene expression inheritance classification and statistical analyses

A total of 35,883 genes were detected in the RNA-Seq data, however, only 29,946 of these ($\approx 83\%$) were consistently expressed in all accessions and thus were studied here. Both, raw and curated data, as well as a set of functions to data-mine the transcriptomes were gathered into a computer tool called “*Salsa*”^31^, programed in the R^32^ environment. Our R package *Salsa* is publicly available in^33^.

**Figure 1:**
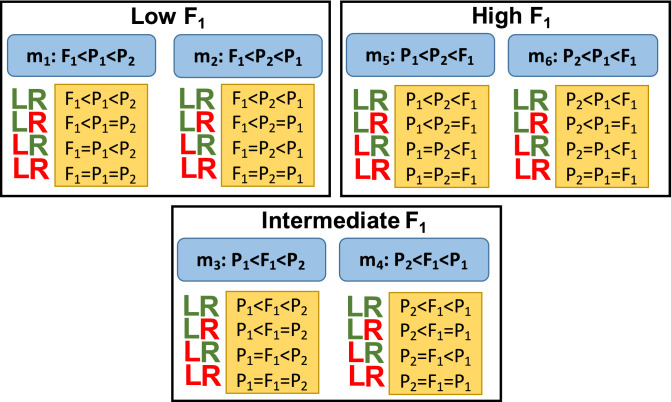
Inheritance class, model and sub-model. Inheritance classes are presented in main rectangles (Low $F_1$, Intermediate $F_1$ and High $F_1$); models ($\mathbf {m}_1$ to $\mathbf {m}_6$) are presented in blue rectangles within each class and sub-models are presented in the orange rectangles below each model. Adjacent to orange rectangle the symbols “**L**” and “**R**” represent the left and right hand side inequality of the corresponding model ($\mathbf {m}_i$). In those letters, green means that the corresponding inequality stands, while red means that the inequality is substituted by equality. The final result of each row (sub-model) is shown in the orange rectangle.

Here we denote standardized gene expression for a gene by the same symbol used for the genotype, say, $P_1,\ P_2$ and $F_1$. To classify inheritance, we will consider the order in which these numbers can be sorted. First, noticing the order of the $F_1$ with reference to their parents, we obtain three classes: “Low $F_1$” –when the value of the $F_1$ is lower than either of the two parents; “Intermediate $F_1$”—when the $F_1$ is within the parent’s values and “High $F_1$”—when the value of the $F_1$ is higher than either of the two parents. The main rectangles in Fig. 1 show the three classes within the main rectangles.

By taking into account the order of the parents, $P_1$ and $P_2$, within each one of the three classes we obtain a further classification of “models”, denoted as $\mathbf {m}_i;\ i = 1, 2, 3, 4, 5$ and 6, and represented within the blue rectangles in Fig. 1; for example, class “Low $F_1$” is divided into models $\mathbf {m}_1: F_1< P_1 < P_2$ and $\mathbf {m}_2: F_1< P_2 < P_1$, etc. Up to here we exhaustively classified inheritance into three classes that imply 6 different models, simply by observing the values of standardized gene expression in the cross participants, but without employing any statistical test.

However, we must perform statistical tests in order to decide if the inequalities postulated between pairs of gene expressions at each one of the $\mathbf {m}_i$ models are significant or not. Table 2 presents the possible tests as well as their null hypotheses.

**Table 2:** Formal hypotheses definitions for pairs of cross participants.

Pair	${\mathcal {H}}_0$
$P_1, P_2$	$P_1 = P_2$
$P_1, F_1$	$P_1 = F_1$
$P_2, F_1$	$P_2 = F_1$

Take as an example the model $\mathbf {m}_1: F_1< P_1 < P_2$, which belongs to the “Low $F_1$” class and is presented in the upper left hand side blue rectangle in Fig. 1. This model arises when we observe that a particular gene has the lowest expression in $F_1$, followed by the value in $P_1$ and then by the highest value in $P_2$. Assume that the observed order is correct. Then we still need to test if in fact the values of $F_1$ and $P_1$ (the left hand side of the $F_1< P_1 < P_2$ inequality) are different or not by testing the hypothesis $P_1, F_1$ on Table 2, and also we need to corroborate if the values of $P_1$ and $P_2$ (the right hand side of the $F_1< P_1 < P_2$ inequality) are different or not by testing the hypothesis $P_1, P_2$ on Table 2. From the four possible results of these two test we have the four sub-models presented in the orange rectangle below the blue rectangle containing model $\mathbf {m}_1: F_1< P_1 < P_2$ in Fig. 1. Those sub-models are $F_1< P_1 < P_2$, $F_1 < P_1 = P_2$, $F_1 = P_1 < P_2$ and $F_1 = P_1 = P_2$ and result from rejecting (green letter) or not rejecting (red letter) the hypotheses about the “left” (**L**) hand side participants –in this case $F_1$ and $P_1$, and also rejecting or not the “right” (**R**) hand side participants—in this case $P_1$ and $P_2$. For example, sub-model $F_1 < P_1 = P_2$, results when we observed model $\mathbf {m}_1: F_1< P_1 < P_2$, and rejected the null hypothesis ${\mathcal {H}}_0: P_1 = F_1$, but fail to reject the null hypothesis ${\mathcal {H}}_0: P_1 = P_2$. This results in the annotation with a green **L** followed by a red **R** in the corresponding sub-model in Fig. 1.

Note that each one of the six orange rectangles in Fig. 1 contains the four different sub-models for a given model (blue rectangle), making a total of $6 \times 4 = 24$ sub-models. Nevertheless, in all cases when the tests for the left and right hand side participants are not rejected (rows annotated with red letters **LR**; last rows at each orange rectangle) the sub-model is always the one in which all three participants have statistically the same expression, and those six sub-models can be written as $P_1 = F_1 = P_2$ by the property of equality’s transitivity. Thus we have in fact not 24, but $24-5=19$ different sub-models, and sub-model “$P_1 = F_1 = P_2$” is considered to be the “null model”.

In all the six orange rectangles presenting sub-models in Fig. 1, the first row presents a sub-model equal to the corresponding model. Those are the case annotated with green **LR** letters—cases where both hypotheses were rejected, and we call those cases “main significant sub-models” in posterior analyses.

Given that we have a time course experiment with 7 times points sampled along fruit development ($0, 10, \cdots , 60$ DAA), the three hypotheses shown in Table 2 must be tested for each one of those time points. To obtain the *p*-values for each test at each time and in crosses $\mathbf {C}_1$ and $\mathbf {C}_2$ (see Table 1), we used the exactTest function of the R package “*edgeR*”^34^. Then the *p*-values were transformed to *q*-values by the method in^35^ to obtain a suitable False Discovery Rate (FDR) of approximately 5% (see details in Supplementary S1).

In summary, for each cross we used a hierarchical classification of inheritance which is shown in Fig. 1. In a first step we observed the relative position of the $F_1$ with reference to both parents, obtaining classes “Low $F_1$”, “Intermediate $F_1$” and “High $F_1$” (main rectangles in Fig. 1). On a second step we determined by observation the order of the two parents within each class, obtaining two models for each class—those models are shown as blue rectangles within each class in Fig. 1. Finally, in a third step we took into account the results of the statistical tests performed to subdivide each model into the four possible sub-models shown in the orange rectangles in Fig. 1. We end up with an exhaustive classification of 19 inheritance patterns, represented as sub-models.

To test independence of classification criteria in contingency tables—resulting from counting frequencies of inheritance types, we used the log likelihood ratio test or “G-test”^36^. With this tool we tested randomness and consistency in time of inheritance patterns. Details of these analyses are given in Supplementary S2.

We used Gene Ontology (GO)^37^ enrichment analysis for all the $19 \times 7 = 133$ sets of genes resulting from the combinations of the 19 sub-models and 7 times of development. These analyses were performed for aspects “Biological Process” (BP), “Molecular Function” (MF) and “Cell Component” (CC), with an initial FDR of 10%. See Supplementary S3 for details.

All analyses were performed with the data and functions implemented in our *Salsa*^31^ package. Statistical results were gathered into a set of R objects, which were documented and deposited in a public repository^38^ (See Supplementary S4).

### R package

Curated data and functions are deposited as an R package (“*ChiliCross*”)^38^, available at (Link: https://zenodo.org/record/5119746#.YPhQXZOuIcg).

### Plant collection

Seeds for the parent accessions, $\mathbf {C}_1$ (CM) and $\mathbf {C}_2$ (QU), were obtained from the seed bank of the “Instituto Nacional de Investigaciones Forestales, Agrícolas y Pecuarias” (INIFAP), México. $F_1$ crosses of the parents, CQ and QC (see Table 1), were performed in our laboratory. All plants were grown as previously described in^26^ and we confirm that all plant materials used in this study comply with the relevant institutional, national, and international guidelines and legislation.

## Results

### Inheritance of gene expression overall times of fruit development

Of the 29,946 genes consistently expressed in all accessions, 22,374 ($\approx 75\%$) were expressed in frequencies $>0$ in at least one of the participants in the crosses (accessions CM, QU, CQ and QC; see Table 1). Because we have 2 crosses and 7 times of development, we will be studying a total of 22,374 $\times 2 \times 7=$ 313,236 cases of inheritance. However, of these 313,236 cases 3,440 ($\approx 1\%$) have an observed expression equal to zero in the three participants of a cross. Those cases represent particular instances of the null sub-model “$P_1=F_1=P_2$”, i.e., cases where $P_1=F_1=P_2=0$.

To obtain a general panorama of the inheritance of gene expression, we classified all patterns into classes, models and sub-models as shown in Fig. 1 without taking into account the time of fruit development.

**Figure 2:**
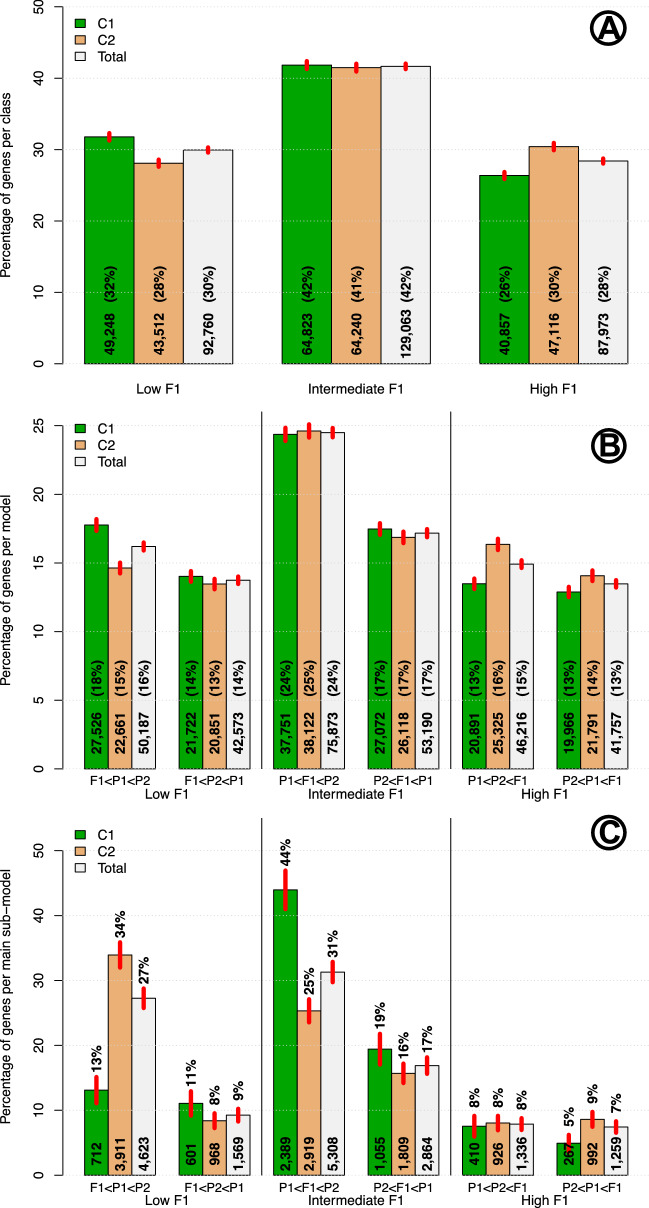
Frequency of class, model and main sub-model per cross over all times of fruit development. Colored bars give percentages for cross $\mathbf {C}_1$, $\mathbf {C}_2$ and the Total of both crosses. Red lines in the middle of the top of bars are Confidence Intervals (CI) for percentages with $\alpha =1 \times 10^{-5}$. Numbers in bold at the base of each bar give raw number of cases. Rounded percentages are given in bars in panels **(A,B)**, and above bars in panel **(C)**. Panel **(A)** Frequency per class; Panel **(B)** Frequency per model; Panel **(C)** Frequency per main significant sub-model.

Panel (A) in Fig. 2 shows bar plots of percentages of cases per class. Without taking into account the cross, i.e., in the grey bars for the total of both crosses, we see that the proportion of low, intermediate and high $F_1$ classes are 30, 42 and 28%, respectively; thus, the cases where the $F_1$ has a value intermediate to the ones in the parents is the most prevalent. Confidence intervals (CI’s) for these percentages—represented by vertical red lines in the top of the bars, show that there are significant ($p<1 \times 10^{-5}$) differences between crosses for classes “Low $F_1$” and “High $F_1$”. The “Low $F_1$” is higher by $\approx 4\%$ in cross $\mathbf {C}_1$ compared with cross $\mathbf {C}_2$ (values of $\approx$ 32 and 28% respectively), while the reverse happens for the proportions in the class “High $F_1$”. For that class we have percentage higher in $\mathbf {C}_2$ ($\approx$ 30%) compared with the percentage of $\mathbf {C}_1$ ($\approx$ 26%). These differences imply that there is a clear effect of the sex of the parent in the proportion of the “Low $F_1$” and “High $F_1$” classes, while that effect is not strong for the “Intermediate $F_1$” class, where both percentages are close to 41%, with a difference between crosses of $\approx 1\%$.

Panel (B) in Fig. 2 presents the percentages of cases for the six models ($\mathbf {m}_i; i = 1, 2, \cdots 6$; blue rectangles in Fig. 1) within the three classes. This panel allow us to analyze with more detail than in Panel (A) the inheritance patterns per cross and in total. Within class “Low $F_1$” we see that in total the pattern “$F_1<P_1<P2$” has a significantly ($p<1 \times 10^{-5}$) larger percentage ($\approx 16\%$) than the pattern “$F_1<P_2<P1$” ($\approx 14\%$), and the differences in percentages of “$F_1<P_1<P2$” between the two crosses are larger than the differences in percentages of “$F_1<P_2<P1$” between the two crosses. On the other hand, within the class “Intermediate $F_1$” model “$P_1<F_1<P_2$” ($\approx 24\%$) is preponderant with regard to the model “$P_2<F_1<P_1$” ($\approx 17\%$); however, there are not high differences between crosses for those two models. Finally, models “$P_1<P_2<F_1$” and “$P_2<P_1<F_1$” within class “High $F_1$” present heterogeneity both, between models and crosses, with percentages that vary between $\approx 13\%$ and $\approx 16\%$. In summary, panel (B) shows that different models present heterogeneous proportions that in various cases differ between crosses.

It is important to remember that in panels (A) and (B) of Fig. 2 the results are purely observational; i.e., percentages of classes and models are presented directly as observed, without performing any statistical tests. In contrast, panel (C) in Fig. 2 presents percentages in the “main significant sub-models”, i.e., sub-models that are equal to the corresponding models but statistically significant. Thus, while in panel (B) the percentages refer to the total number of cases, in panel (C) such percentages are exclusively for genes that present the corresponding main significant sub-models after performing the statistical tests, having a FDR of $\approx 5\%$. As in panel (B), in panel (C) percentages over all the 6 models per cross add to 100%, however, as can be seen by the numbers in each one of the bars, the numbers of main significant sub-models in panel (C) are always a small fraction of the same model presented in panel (B). For example, while the number of cases with model “$F_1<P_1<P2$” is 27,526 in panel (B), it is only 712 in panel (C). This is because, after observing all the 27,526 cases of model “$F_1<P_1<P2$”, we performed statistical tests to re-classify these cases into the four possible sub-models: “$F_1<P_1<P2$”, “$F_1<P_1=P2$”, “$F_1=P_1<P2$” and “$F_1=P_1=P2$” (see orange rectangle in upper left hand side corner of Fig. 1), and only in 712 cases both statistical tests, $P_1=F_1$ and $P_1=P_2$ (Table 2) were rejected. Details for the 19 sub-models are presented in Supplementary S1.

In panel (C) of Fig. 2 we see that the percentages of the six main significant sub-models are heterogeneous; nevertheless, standing out by having the highest percentages are the significant sub-models “$P_1<F_1<P_2$” in $\mathbf {C}_1$ with $\approx 44\%$ and “$F_1<P_1<P_2$” in $\mathbf {C}_2$ with $\approx 34\%$ of the totals per cross. Interestingly, the main significant sub-models with lower percentages are the ones in class “High $F_1$”, with values that range between 5 and 9%.

**Figure 3:**
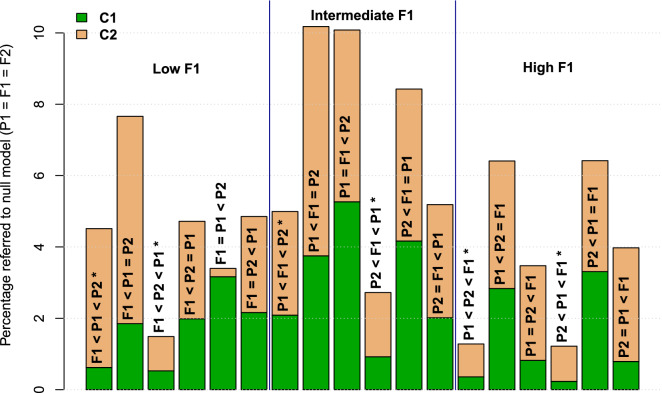
Percentages of sub-models per cross with reference to the total number of null models ($\mathbf {P}_{\mathbf {1}} = \mathbf {F}_{\mathbf {1}} = \mathbf {F}_{\mathbf {2}}$). Sub-model identified in or over each one of the bars. The six main significant sub-models (that contain only inequalities) are identified with an asterisk.

After statistical analyses to determine significant sub-models (presented in the orange rectangles in Fig. 1), a total of 114,436 and 100,474 combinations of genes $\times$ times of development in $\mathbf {C}_1$ and $\mathbf {C}_2$, respectively, were classified as the null model $P_1 = F_1 = P_2$. To appreciate the differences in frequencies of the other 18 sub-models in each one of the two crosses, Fig. 3 presents the percentages of such sub-models per cross, with reference to the total number of null models.

In Fig. 3 we can notice, by the height of the whole bars, that the percentage of sub-models with reference to the null model varies between approximately 1.2 and 10.2%. Even when the proportions of the sub-models are heterogeneous and different for each cross, there is a significant ($p \approx 0.03$) linear correlation of $\hat{r} \approx 0.53$ between the percentages per cross. For $\mathbf {C}_1$ the sub-model with larger frequency is $P_1=F_1<P_2$ with $\approx 5.3\%$, while for $\mathbf {C}_2$ that place corresponds to sub-model $P_1<F_1=P_2$ with $\approx 6.4\%$. These two models belong to the class “Intermediate $F_1$”, which as we saw in Fig. 2 is the most frequent class. On the other hand, the two less frequent sub-models are $P_2<P_1<F_1$ in $\mathbf {C}_1$ with $\approx 0.2\%$ and $F_1=P_1<P_2$ in $\mathbf {C}_2$ also with $\approx 0.2\%$. These less frequent sub-models belong to the extreme classes “High $F_1$” and “Low $F_1$”, respectively.

### Inheritance of gene expression during fruit development

**Figure 4:**
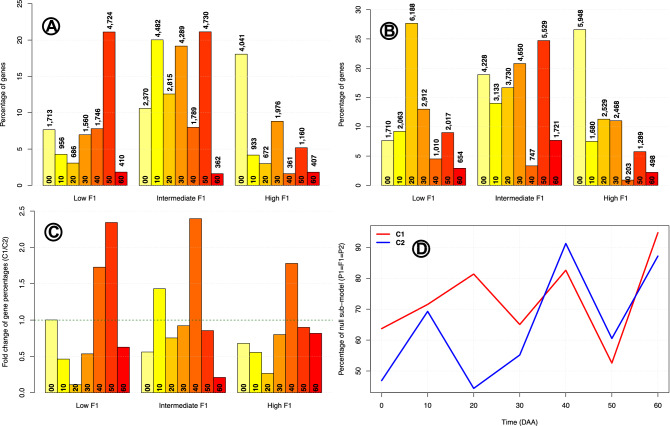
Frequency of class per time of fruit development. In panels (**A–C**) bars present values per class (label in *X*-axis) and time of development (at bottom of each bar). Null sub-model ($P_1 = F_1 = F_2$) is not included. Panels (**A,B**) give percentages of genes per class $\times$ time combination for crosses $\mathbf {C_1}$ and $\mathbf {C_2}$, respectively. Number of genes are presented over each bar. Panel (**C**) gives fold change for percentages $\mathbf {C_1} / \mathbf {C_2}$ for each class $\times$ time combination; the green line at $Y=1$ denotes equality of proportion. Panel (**D**) gives percentages of null sub-model ($P_1 = F_1 = F_2$) in *Y*-axis as function of time of development in *X*-axis per cross.

As explained before, by taking into account statistical considerations, i.e., significance of the tests performed, we exhaustively classified genes into 19 inheritance sub-models. It is important to remember that the null sub-model, “$P_1 = F_1 = F_2$”, is assigned to a given gene within a time of development either, when the expression in the three participants of the cross is equal to zero, or when the corresponding tests between the mean were not significant. As a result of these conditions, the most frequent sub-model over all times of development was the null one, with a frequency of 114,436 cases ($\approx 73\%$) in cross $\mathbf {C}_1$ and 100,474 ($\approx 65\%$) in cross $\mathbf {C}_2$. Previously we showed results without taking into account the time of fruit development. Figure 4 displays the frequency of classes when the time of fruit development is considered.

Panel (A) in Fig. 4 is a bar plot for the percentages of genes per class (“Low $F_1$”, “Intermediate $F_1$” and “High $F_1$”) and time of fruit development ($00, 10, \cdots , 60$ DAA –label at the bottom of each bar) in cross $\mathbf {C_1}$, excluding the null model. Numbers above each bar give the number of genes. In this plot we can see that the time of development strongly influences inheritance class; i.e., the percentages and raw gene numbers are highly variable per time within each class. For example, “Low $F_1$” class is preponderant at 50 DAA by being present in 4,724 genes, but at time 60 DAA the number of genes with this class is only of 410. Panel (B) in Fig. 4 is as panel (A) but for cross $\mathbf {C_2}$, and there we can see also high heterogeneity of frequencies of the three classes along the times of development.

To compare frequencies of genes per class and time of development, panel (C) in Fig. 4 presents the fold changes of the number of genes per combination of class $\times$ time of development of the frequency in $\mathbf {C_1}$ over the corresponding frequency in $\mathbf {C_2}$. Equality of frequencies in the two crosses happens if this fold change is equal to 1, a point marked by the green horizontal line. We can see that only in class “Low $F_1$” at 0 DAA the fold change is $\approx 1$, signaling equality of frequencies. In all other cases the observed fold changes are highly different from the neutral value of 1; in two cases, “Low $F_1$” at 50 DAA and “Intermediate $F_1$” at 40 DAA the fold changes are larger than 2, indicating that the frequency in $\mathbf {C_1}$ is more than twice times the frequency than in $\mathbf {C_2}$, and conversely, in 4 cases, say “Low $F_1$” at 10 DAA, “Low $F_1$” at 20 DAA, “Intermediate $F_1$” at 60 DAA and “High $F_1$” at 20 DAA the fold is smaller than 0.5, indicating that the frequency in $\mathbf {C_2}$ is more than twice times the frequency than in $\mathbf {C_1}$. In general, these differences in fold change indicate that the cross of origin highly influence inheritance pattern.

Panel (D) in Fig. 4 presents the plot of the percentages of the null sub-model, $P_1 = F_1 = F_2$, per time of development in each cross. Those percentages vary from a minimum of $\approx 45\%$ for cross $\mathbf {C_2}$ at 20 DAA, up to a maximum $\approx 95\%$ for cross $\mathbf {C_1}$ at 60 DAA, with a median of $\approx 67\%$. Given that the power of the tests for each inheritance sub-model at each time of development is approximately equal, this heterogeneity in the percentages of cases where the three participants of the crosses have statistically equal gene expression (sub-model $P_1 = F_1 = F_2$), indicates that the dynamics of inheritance patterns change with both, cross and time of development.

To evaluate the relative importance of the factors “cross” ($\mathbf {C}_1$ or $\mathbf {C}_2$) and “time of fruit development” (seven points) on the number of cases of each sub-model, we carried out various statistical analyses. Even when the number of cases of each sub-model is differentially influenced by cross and time, in general the cross of origin is approximately 3.8 times more important than the time of development in determining the number of sub-models (see Supplementary S2).

In summary, frequencies of gene expression sub-model are influenced by both, the direction of the cross as well as by the time of development. Even when for a given gene sub-models can change through fruit development, there is a strong and significant tendency of the genes to have a consistent inheritance pattern over development; details of this fact are presented in Supplementary S2.

### Gene Ontology (GO) enrichment analyses

GO enrichment analysis was performed within the *Salsa* package^31,33^ for all the 133 gene sets resulting from the combinations of the 19 sub-models and 7 times of development for each one of the two crosses. Full results are gathered into the “*ChiliCross*”^38^ R package (see Supplementary S4). We used an initial FDR of 10% for the analyses and Table 3 presents the raw numbers and percentages of enriched terms found for each aspect in each one of the crosses.

**Table 3:** Number of GO terms significant at 10% FDR in aspects “Biological Process” (BP), “Molecular Function” (MF) and “Cell Component” (CC). Values inside parenthesis are approximated percentages of the total.

Cross	BP	MF	CC	Total
$\mathbf {C}_1$	1,762 (27)	1,231 (19)	356 (5)	3,349 (51)
$\mathbf {C}_2$	1,719 (26)	1,177 (18)	377 (6)	3,273 (49)
Total	3,481 (53)	2,408 (36)	733 (11)	6,622 (100)

In Table 3 we see that the total number of enriched terms is roughly the same for both crosses, comprising approximately 51 and 49% of the total for crosses $\mathbf {C}_1$ and $\mathbf {C}_2$, respectively. On the other hand, the approximate percentages for the aspects are 53, 36 and 11% for BP, MF and CC, respectively. By taking into account that GO enrichment was performed for 133 gene sets per cross, we obtained a median of approximately 9 terms enriched for each individual analysis performed. This demonstrates that particular inheritance sub-models, found at specific times of fruit development, have sets of genes preferentially involved into particular BP, with distinct MF and related with specific CC.

#### Biological Process (BP) enrichment

Of the total number of 3,481 BP results obtained (Table 3), 3,096 (89%) have an odd value larger than one, and thus present in fact a significant gene enrichment, while the remaining 385 (11%) have an odd value smaller than one, and thus the gene set is depleted of the corresponding term. Here we are going to study only the 3,096 cases of enrichment.

The 3,096 enrichment cases are heterogeneously distributed among the 133 combinations of sub-model $\times$ development time. The most frequent sub-model over all times was, by much, the null one ($P_1=F_1=P_2$), with a total of 2,572 cases, i.e., 83% of the total. Because the null model implies equality of expression in the three participant of each cross, those enrichment cases in fact refer to enrichment at particular times of development. On the other hand, the remaining $3,096-2,572=524\ (17\%)$ cases occur in one of the 18 not null sub-models, distributed among different development times. The number of cases in particular combinations of not null sub-model $\times$ times of development varies between a minimum of 0 (in 74 of the 126 combinations) and a maximum of 68, with a mean of 4.159 cases per combination. The combination with the largest number of enriched terms (68) occurs for the sub-model $F_1<P_2=P_1$ at time 20 DAA.

In the 3,096 enrichment analyses there are 580 different GO BP terms, of which 176 (30%) are unique, i.e., present in a single sub-model $\times$ time combination, and the remaining 404 (70%) are shared by two or more of those combinations. Supplementary S3 presents the bi-variate distributions of the numbers of GO BPs.

**Figure 5:**
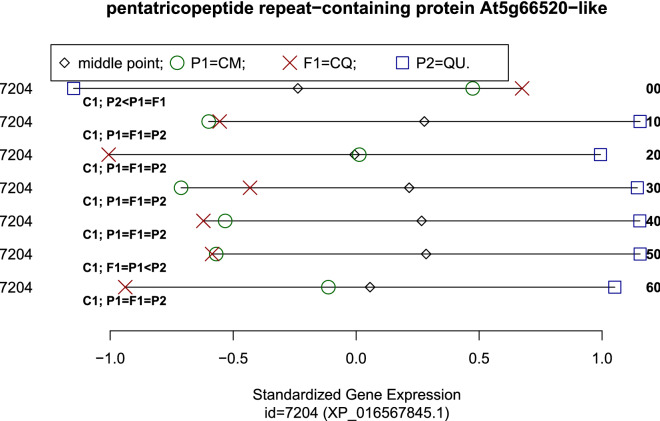
Standardized gene expression (*X*-axis) for gene coding protein XP_016567845.1 at each time of development (rows in *Y*-axis). Left margin presents gene id while right margin gives the time of development in DAA. Each row is annotated with the cross ($\mathbf {C}_1$), followed by the sub-model.

An interesting example of an enriched BP is given by the term “*negative regulation of growth*” (GO:0045926). All three genes annotated in this BP in *Capsicum* present the sub-model $P_2 < P_1 = F_1$ at the time of fruit development 0 DAA, and the *p*-value for the enrichment analysis was $\approx 0.0004$, reaching a FDR $<8\%$. The three genes annotated in BP “negative regulation of growth” are pentatricopeptide repeat-containing proteins, with *Arabidopsis* orthologs AT5G66520, AT1G59720 and AT2G46050. Figure 5 presents a plot for the standardized gene expression of the first gene annotated in the BP “negative regulation” of growth, which is the gene coding for protein XP_016567845.1, ortholog to AT5G66520.

Figure 5 is an example of running the R function “cross.plot()”, that we developed to analyze the data with the package “*ChiliCross*”^38^. That function can be used to plot and interpret the inheritance patterns of any set of seven cases (combinations of gene $\times$ time of development) for the almost 30,000 genes studied, and thus represents a powerful interpretation tool for inheritance patterns (see Supplementary S4 for details).

To interpret plots given by the function “cross.plot()”, it is necessary to keep in mind that the *X*-axis shows always *standardized* gene expressions for the participants of the cross, defined by the upper left hand side legend of the plot. Thus, the vectors formed by the mean expression of parents and $F_1$ have always an average of zero and a standard deviation of one, representing the **model** of inheritance of a given gene id (annotation in the left had side margin) at a particular time of development (annotation in the right had side margin). Below the line that expands along the standardized gene expression in each row, we have an annotation corresponding to the **sub-model** –which takes into account the significance of the differences observed.

For example, the first row in Fig. 5, corresponds to the time 00 DAA, and has annotation “**C1**; $\mathbf{P2} <\mathbf{P1} =\mathbf{F1}$”, meaning that the plot shows results from cross $\mathbf {C}_1$ and that the sub-model estimated for that gene at that cross is $P_2<P_1=F_1$; i.e., even when we see that the observed values are in the order $P_2<P_1<F_1$, the difference between $P_1$ and $F_1$ was not-significant. Interpretation for the other six rows in Fig. 5 (times $10, 20, \cdots , 60$ DAA) is analogous to the one for the first row (time 00). In Fig. 5 we can see how the standardized expression of the gene changes through the time of development, and the annotations below each row tell us which changes were significant, implying a change in sub-model. In rows 2, 3, 4 and 5 in Fig. 5, corresponding to times 10, 20, 30 and 40, we see changes in the observed values of mean inheritance in the participants; however, in those four rows the annotation is the same: “**C1**; $\mathbf{P1} =\mathbf{F1} =\mathbf{P2}$”, meaning that the estimated sub-model was the same in the four cases, i.e., the null sub-model “$P_1 = F_1 = P_2$”. Thus, between 10 and 50 DAA there are not significant changes in gene expression between the participants of the cross $\mathbf {C}_1$ for the gene plotted.

**Figure 6:**
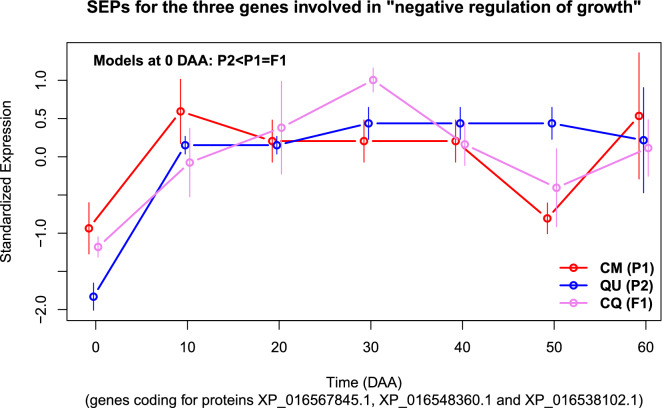
Standardized Expression Profiles (SEPs) for the sets of the three genes annotated in BP “negative regulation of growth” in accessions CM, QU and CQ. Confidence Intervals (CIs) for each SEP at each time are presented as thin vertical lines.

Previously, in^31^, we developed a methodology to estimate, test and plot “Standardized Expression Profiles” (SEPs) for sets of genes. Briefly, a SEP for a set of genes is a numerical vector for the means at each time of development which has a mean of zero and a standard deviation of one. Approximate Confidence Intervals (CIs) for the mean at each time are obtained using the dispersion of individual genes at each time. Figure 6 presents the SEPs for the three genes involved in “negative regulation of growth” in accessions CM, QU and CQ, which correspond to $P_1$, $P_2$ and $F_1$, respectively.

In Fig. 6 we can see how at 0 DAA the set of the three genes annotated in the BP “negative regulation of growth” have a sub-model $P_2 < P_1 = F_1$, i.e. the mean expression of the three genes in $P_2$ (accession QU) is the lowest at 0 DAA, while the mean expression of $P_1$ (accession CM) and the $F_1$ (accession CQ) is higher, and not significantly different between $P_1$ and $F_1$ –because the corresponding CIs are overlapped. It is important to note that the mean of standardized gene expression for these sets of three genes changes through time—a fact that was already observed for one of the three genes in Fig. 5. For example, in Fig. 6 at time 50 DAA, the inheritance of these three genes is reversed with reference to the one at 0 DAA; at 50 DAA we have that $F_1 = P_1 < P2$—a fact that is corroborated for the first gene in Fig. 5.

Figures 5 and 6 illustrate the fact that all GO BP results can be studied in detail by plotting the inheritance patterns of the genes involved—as in Fig. 5, or showing and testing the standardized expression of the sets of genes along time of development—as in Fig. 6. Supplementary S4 shows how to perform such analyses by using the data deposited in^38^.

Table 4 breaks down the 3,096 cases of enriched GO BPs into classes to facilitate interpretation.

**Table 4:** Number of GO terms enriched in “Biological Process” (BP) per class.

Cross	Low $F_1$	Intermediate $F_1$	High $F_1$	$P_1=F_1=P2$	Shared	Total
$\mathbf {C}_1$	26	17	9	543	979	1,574
$\mathbf {C}_2$	30	29	10	445	1,008	1,522
Total	56	46	19	988	1,987	3,096

In Table 4 columns “Low $F_1$”, “Intermediate $F_1$” and “High $F_1$” present the numbers of enriched GO BPs which are exclusive to those classes, column“$P_1=F_1=P_2$” gives the numbers of enriched GO BPs in instances of the null sub-model, while column “Shared” presents the number of enriched GO BPs that are shared by more than one class and time combination. From this table we can see that there are not strong differences between the numbers of enriched GO BPs between crosses, and also that the largest numbers of those processes are shared in more than one class, followed by the ones present in the null sub-model.

The degree of enrichment in a GO analyses is measured by the “odds” from the 2 $\times$ 2 contingency table analyzed. In this context, the odds estimate how more likely is to find a gene annotated with the process analyzed in the target set of genes, compared with the probability of finding it in the set of genes not annotated with the process. When all genes annotated with the given process are in the target set, the value of the odds become “infinite” (*x*/0), denoting that it is impossible to have a larger enrichment, and that value is denoted by the symbol “$\infty$”. Table 5 presents the GO BPs with larger odd values for each one of the combinations of class $\times$ cross presented in Table 4.

**Table 5:** Enriched “Biological Process” (BP) with the highest odds per combination of class $\times$ cross.

Row	Class	Cross	Sub-model	time	GO	Description	Odds
1	Low $F_1$	$\mathbf {C}_1$	$F_1<P_2=P_1$	00	GO:0015758	Glucose transport	86
2	Low $F_1$	$\mathbf {C}_2$	$F_1<P_1<P_2$	00	GO:0046500	S-adenosylmethionine metabolic process	62
3	Inter. $F_1$	$\mathbf {C}_1$	$P_1<F_1=P_2$	50	GO:0010109	Regulation of photosynthesis	27
4	Inter. $F_1$	$\mathbf {C}_2$	$P_2=F_1<P_1$	20	GO:0010466	Negative regulation of peptidase	95
5	High $F_1$	$\mathbf {C}_1$	$P_1<P_2<F_1$	50	GO:0018149	Peptide cross-linking	421
6	High $F_1$	$\mathbf {C}_2$	$P_1<P_2<F_1$	50	GO:0018149	Peptide cross-linking	805
7	$P_1=F_1=F_1$	$\mathbf {C}_1$	$P_1=F_1=P_2$	60	GO:0040029	Epigenetic regulation of gene expression	$\infty$
8	$P_1=F_1=F_1$	$\mathbf {C}_2$	$P_1=F_1=P_2$	60	GO:0040029	Epigenetic regulation of gene expression	$\infty$
9	Shared	$\mathbf {C}_1$	$P_1=F_1=P_2$	60	GO:0046834	Lipid phosphorylation	$\infty$
10	Shared	$\mathbf {C}_2$	$P_1=F_1=P_2$	40	GO:0046834	Lipid phosphorylation	$\infty$

In rows 1 to 4 in Table 5 we can see that the GO BPs with the largest odds for classes “Low $F_1$” and “Inter. $F_1$” (intermediate $F_1$) are different GO BPs that happen for different sub-models and times in both independent crosses, thus those enriched BPs are cross and time dependent. In contrast, rows 5 to 10 in that table repeat the same GO BP with the same sub-model and, in all cases except for row 10, at the same time of development. This implies that such BPs are consistently highly enriched, independently of the cross and almost independently of the time of development. Supplementary S3 presents an additional table of enriched BPs per time $\times$ pattern combinations.

#### Molecular Function (MF) enrichment

As seen in Table 3, we have a total of 2,408 significant cases for MF. However, of those cases only 1,772 have odds $> 1$, and thus are truly “enriched”. Table 6—which is homologous to Table 4 but for MF, presents the numbers of enriched GO MF per class, while Table 7 presents the MF for class and cross that gave the highest value of odds within each combination.

**Table 6:** Number of GO terms enriched in “Molecular Function” (MF) per class.

Cross	Low $F_1$	Intermediate $F_1$	High $F_1$	$P_1=F_1=P2$	Shared	Total
$\mathbf {C}_1$	14	27	8	357	530	936
$\mathbf {C}_2$	22	24	3	319	468	836
Total	36	51	11	676	998	1,772

In Table 6 we can see that cross $\mathbf {C}_1$ has a higher number of enriched MF, 936 (53%), than $\mathbf {C}_2$, which has 836 (47%). We can also note that the numbers of enriched MF exclusive of each class, excluding the ones in $P_1=F_1=F_1$ (676; 38%), are in decreasing order 51 (3%) for “Intermediate $F_1$”, 36 (2%) for “Low $F_1$” and only 11 (1%) for the “High $F_1$”.

**Table 7:** Enriched “Molecular Function” (MF) with the highest odds per combination of class $\times$ cross.

Row	Class	Cross	Sub-model	time	GO	Description	Odds
1	Low $F_1$	$\mathbf {C}_1$	$F_1=P_1<P_2$	50	GO:0003714	transcription corepressor activity	57
2	Low $F_1$	$\mathbf {C}_2$	$F_1<P_1=P_2$	30	GO:0008685	cyclodiphosphate synthase activity	$\infty$
3	Inter. $F_1$	$\mathbf {C}_1$	$P_2<F_1=P_1$	10	GO:0003785	actin monomer binding	$\infty$
4	Inter. $F_1$	$\mathbf {C}_2$	$P_1<F_1=P_2$	50	GO:0004655	porphobilinogen synthase activity	$\infty$
5	High $F_1$	$\mathbf {C}_1$	$P_1<P_2<F_1$	50	GO:0003810	protein-glutamine gamma-glutamyltransferase activity	$\infty$
6	High $F_1$	$\mathbf {C}_2$	$P_1<P_2<F_1$	50	GO:0003810	protein-glutamine gamma-glutamyltransferase activity	$\infty$
7	$P_1=F_1=F_1$	$\mathbf {C}_1$	$P_1=F_1=P_2$	20	GO:0019838	growth factor binding	$\infty$
8	$P_1=F_1=F_1$	$\mathbf {C}_2$	$P_1=F_1=P_2$	60	GO:0019838	growth factor binding	$\infty$
9	Shared	$\mathbf {C}_1$	$P_1=F_1=P_2$	00	GO:0015097	mercury ion transmembrane transporter activity	$\infty$
10	Shared	$\mathbf {C}_2$	$P_1=P_2<F_1$	50	GO:0005136	interleukin-4 receptor binding	574

In Table 7 we can see that in 8 of the 10 cases presented, the enrichment level is the highest that can be reached, presenting values of $\infty$ for the odds, and thus denoting that in those cases all genes annotated in the corresponding GO MF were present in the corresponding set of target genes. Also, in Table 7 we can note that for classes “High $F_1$” and null model ($P_1=F_1=F_1$), the same MF was the one with the highest odd value, “protein-glutamine gamma-glutamyltransferase activity” for “High $F_1$” in both crosses at the same time (50 DAA), and “growth factor binding” for $P_1=F_1=F_1$ at times 00 and 50 DAA for $\mathbf {C}_1$ and $\mathbf {C}_2$, respectively.

#### Cell Component (CC) enrichment

As seen in Table 3, we have a total of 733 cases for MF; however, of these only 374 are enriched by having odd values $> 1$. Table 8—homologous to tables 4 and 6 but for CC, presents the numbers of enriched GO CC per class, while Table 9 presents the CC for class and cross with the highest odd value within each combination.

**Table 8:** Number of GO terms enriched in “Cell Component” (CC) per class.

Cross	Low $F_1$	Intermediate $F_1$	High $F_1$	$P_1=F_1=P2$	Shared	Total
$\mathbf {C}_1$	5	6	1	75	88	175
$\mathbf {C}_2$	9	4	2	86	98	199
Total	14	10	3	161	186	374

In Table 8 we can see that cross $\mathbf {C}_2$ surpass $\mathbf {C}_1$ in the number of enriched CC by 24 cases. On the other hand, the number of CC exclusively enriched in classes low, intermediate and high $F_1$—the first three columns of the table, are small: 14, 10 and 3, respectively.

**Table 9:** Enriched “Cell Component” (CC) with the highest odds per combination of class $\times$ cross.

Row	Class	Cross	Sub-model	time	GO	Description	Odds
1	Low $F_1$	$\mathbf {C}_1$	$F_1<P_1<P_2$	00	GO:0044421	extracellular region part	4
2	Low $F_1$	$\mathbf {C}_2$	$F_1<P_1=P_2$	00	GO:0044421	extracellular region part	4
3	Inter. $F_1$	$\mathbf {C}_1$	$P_1<F_1<P_2$	30	GO:0044815	DNA packaging complex	16
4	Inter. $F_1$	$\mathbf {C}_2$	$P_1<F_1=P_2$	50	GO:0009654	photosystem II oxygen evolving complex	11
5	High $F_1$	$\mathbf {C}_1$	$P_2=P_1<F_1$	50	GO:0098800	inner mitochondrial membrane protein complex	9
6	High $F_1$	$\mathbf {C}_2$	$P_1=P_2<F_1$	00	GO:0043228	non-membrane-bounded organelle	2
7	$P_1=F_1=F_1$	$\mathbf {C}_1$	$P_1=F_1=P_2$	00	GO:0017053	transcriptional repressor complex	$\infty$
8	$P_1=F_1=F_1$	$\mathbf {C}_2$	$P_1=F_1=P_2$	00	GO:0017053	transcriptional repressor complex	17
9	Shared	$\mathbf {C}_1$	$P_2=F_1<P_1$	10	GO:0048046	apoplast	73
10	Shared	$\mathbf {C}_2$	$P_1<P_2<F_1$	10	GO:0048046	apoplast	50

In Table 9 we have that the CC “extracellular region part” (rows 1 and 2), “transcriptional repressor complex” (rows 7 and 8) and “apoplast” (rows 9 and 10), are repeated as the ones with the highest odd values for classes “Low $F_1$”, “$P_1=F_1=F_1$” and “Shared”, respectively. Such coincidence of enrichment for the three CC in the two fully independent crosses, suggests that the detected cell components are specially important at specific times of development and have a conserved inheritance class.

### Tools to data-mine the results

Omics studies, as the genome-wide estimation of inheritance performed here, process a large amount of information, and every approach reveals only a restricted aspect of the whole data sets^39^. With the aim of expanding the use of our data for potential discoveries, we gathered them into an R^32^ package called “*ChiliCross*”^38^. That package is publicly available in the dataset repository “*zenodo*”^40^.

The package *ChiliCross* contains standardized gene expression for all genes in both crosses ($\mathbf {C}_1$ and $\mathbf {C}_2$), as well as all GO enrichment results at 10% of FDR for BP, MF and CC, in each one of the 133 gene sets resulting from the $19 \times 7 = 133$ combinations of 19 sub-models $\times$ 7 times of fruit development. Complemented with the *Salsa*^33^ R package, which includes the possibility to plot and analyze SEPs for all genes^31^, these tools assemble a powerful platform to study gene expression during fruit development in chili pepper. For example, in^26^, we found a set of genes that strongly differ in gene expression between domesticated and wild accessions, and that partially explain some of the main differences between those kinds of accessions.

## Discussion

In Fig. 1 we proposed a hierarchical method to classify gene expression inheritance in the $F_1$ of any cross. In a first step we allocated inheritance into three disjoint classes: low, intermediate or high $F_1$ (main rectangles in Fig. 1). In a second step we defined two main models within each class (blue rectangles in Fig. 1). Finally, in a third step, we took into account statistical significance to define the 19 exhaustive sub-models in which gene expression inheritance can be classified (orange rectangles in Fig. 1). This classification system for gene expression as a quantitative trait transcends the Mendelian organization of qualitative characters as “dominant”, “recessive” or “codominant”—although, when the value of gene expression in one of the parents surpass the one in the other we could said that the first “dominates” the second, and when the value in the $F_1$ is between the ones of the two parents we could say that the character has “intermediate inheritance”. Notwithstanding, our proposed sub-model system gives a precise mathematical definition to inheritance patterns that goes beyond the description that could be given by single word, and this is not only desirable but necessary by the quantitative nature of gene expression. It is important to underline that the schema of classifying a quantitative character into 19 sub-models is not limited to gene expression, but applicable to any quantitative character. Quantitative traits important for crop production or improvement could be classified with our schema, with the advantage of precisely determining their inheritance before attempting to dissect its nature by a posterior QTL analysis.

Our system of classification of inheritance patterns for gene expression into 19 categories is analogous to the one presented in^11^ for describing the gene expression levels in inbreds and hybrids of maize (Box Text 2 in that reference). However, we consider that our system is more adequate than the one presented there, which makes a classification into only 8 categories, but needs a previous categorization of the parents as “high” or “low” in the measure of expression. In contrast, our system does not need an *a priory* segregations of the parent’s expression—which could be significant or not, and defines all possibilities being fully exhaustive (see Fig. 1 and discussion above). Also, in panel (a) of Figure 3 in^6^, the authors summarize in a box plot the models of gene expression inheritance from^41^, mentioning that gene expression levels in hybrids are not strictly related to the genetic concepts of dominance and overdominance. Even when the schema shown in that figure helps to graphically appreciate some of the differences in relative gene expression between parents and hybrid, it does not give all the possibilities—including statistical equalities, that our classification system does, and thus in some sense over-simplify the classification problem.

Gene expression is influenced by variants located within the promoter or enhancer of the gene, i.e., by “*cis* effects”, and also by “*trans* effects”, which are driven by diffusible elements such as transcription factors located anywhere in the genome^42^. In general, *cis* elements may segregate in linkage with the relevant gene, while *trans* elements could frequently segregate independently^43^. Up to this point our analyses of inheritance patterns cannot discriminate between *cis* and *trans* elements; however, allele-specific expression analyses^44^ could help to differentiate between them. Using that method in *Arabidopsis lyrata* hybrids^45^, showed that in the majority of cases genes with maternal effect expressed both parental alleles, while in^17^ the authors studied inheritance patterns in cabbage, concluding that *cis* effects mediate most of the gene expression divergence in the $F_1$, but also that *trans* factors appear to have a higher effect compared to *cis* elements on parental expression divergence. In chili pepper^46^, performed allele-specific expression analyses in a single cross of a domesticated by a wild accession and their $F_1$ in fruits of 40 DAA. The authors propose that gene expression differences associated to the cultivated form are better explained by *cis*-regulatory hubs acting through *trans*-regulatory cascades. However, the scope of this conclusion is limited by the fact that the study was performed only in one of the two possible directions of the cross and at a single time point of fruit development (40 DAA). As we have seen here, inheritance patterns strongly differ between crosses and vary through fruit development.

The results presented here comprise genome-wide inheritance patterns of gene expression in bidirectional crosses ($\mathbf {C}_1$ and $\mathbf {C}_2$) between two phenotypically contrasting accessions, CM—a domesticated landrace extensively used in chili pepper research and cultivar breeding^28^, and QU, a wild pepper from northcentral México^29^, representing the ancestors of all domesticated *Capsicum annumm* L.^47^. The fact that gene expression was estimated here during the whole fruit development, from mature flower at 0 DAA up to fully mature fruits at 60 DAA, means that we have a comprehensive panorama of the way in which the standardized measure of this parameter behaves.

The first general conclusion of our work is that inheritance patterns of gene expression are heterogeneous and far from a uniform random pattern. In fact, Fig. 2 shows significant departures from the percentages expected under homogeneity of classes, models and sub-models, which are 1/3 ($\approx$ 33%) for each one of the three classes in panel A of Fig. 2, and 1/6 ($\approx$ 17%) for each model and main sub-model in panels B and C of Fig. 2. In Fig. 2 we can see that the preponderant inheritance pattern corresponds to cases where gene expression in the $F_1$ is between the values of the parents, i.e., within class “Intermediate $F_1$”. This is consistent with the results presented in^48^ for *Arabidopsis*, where in the majority of the differentially expressed genes, the hybrids ($F_1$’s) exhibited intermediate expression levels compared with the parents. Also in yeast^49^, report that essential genes are less likely to exhibit an underdominant inheritance pattern, and in *Drosophila* alleles conferring *cis* regulatory variation tend to have an additive influence on gene expression, with the expression level in the $F_1$ being intermediate between those of the two parents^43,50^.

While inheritance classes show approximate percentages of 30, 42 and 28% for low, intermediate and high $F_1$, respectively—total for both crosses in panel A, Fig. 2), variations by model within class in panel B in Fig. 2 demonstrate further heterogeneity of inheritance patterns. For example, in the “Intermediate $F_1$” class, model “$P_1< F_1 < P_2$” is approximately 7% more frequent than model “$P_2< F_1 < P_1$”, showing that when the $F_1$ is intermediate between the two parents, the domesticated parent (CM $= P_1$) tend to have lower expression in more cases than in the inverse cross with wild ancestor (QU $= P_2$), and such difference is consistent in both crosses, $\mathbf {C}_1$ and $\mathbf {C}_2$. On the other hand, the final classification in sub-models, which takes into account statistical significance between cases to reach an approximate FDR of 5%, accentuates the heterogeneity of inheritance patterns differences. For example, in panel C in Fig. 2, we see that main sub-model “$P_1< F_1 < P2$” is estimated in 2,389 cases in $\mathbf {C}_1$, representing the highest percentage for that cross, $\approx 44\%$, but for $\mathbf {C}_2$ the number of cases of such model is 2,919 which represents only $\approx 25\%$ of the cases of main sub-models in that cross, while the highest percentage of the main sub-model in cross $\mathbf {C}_2$ is $\approx 34\%$, which is reached by the 3,911 cases of the main sub-model $F_1< P_1 < P_2$.

In a review of gene expression in the context of maize heterosis^11^, the authors define “allelic variation” as the sequence or regulatory differences found in different parental genotypes and mention that structural differences are are likely to be less important than regulatory ones. We speculate that also in *Capsicum*, the majority of the significant differences in gene expression could be due to regulatory differences, even when the percentages of those differences are larger in our results –panels (B) and (C) in Fig. 2, than the ones reported by that reference in maize, that vary between approximately 5 and 10%. The large number of significant differences observed in expression in our results are possibly due to the large genetic distance between the parents, one of which is a wild and the other a domesticated accession—see Figure 1 in^26^.

In^6^, the authors assert that heterosis-associated gene expression in maize does not present a direct link between classical heterosis hypothesis and gene expression profiles, possibly because of the complex molecular regulation that is required for the manifestation of phenotypical traits. In the context of allele-specific gene expression^6^, cite the work in^41^, mentioning that of 32 genes whose expression in the hybrid deviated from the midparent value, more than half ($18; \approx 56\%$) are regulated by elements in—*cis*. If this is also the case in *Capsicum*, we can infer that a high percentage of the differences observed in our results could be also due to elements in—*cis*. Also in^6^ the authors conclude that detailed expression profiling experiments will further refine the insight about genes differentially expressed between inbred lines and hybrids and, therefore, play a role during heterosis manifestation. On this framework, our results could be useful to disentangle heterosis in chili pepper.

In^7^ the authors performed gene expression analyses in maize inbreds and hybrids with varying levels of heterosis, observing that $\approx 75\%$ of the differentially expressed genes in each hybrid exhibited additive expression patterns, and only a very small percentage exhibited hybrid levels outside the parental range, but the authors also mention that other groups have reported much higher frequencies of hybrid expression outside the parental range in maize. In contrast, in our analyses a very high percentage of genes presents an $F_1$ with significantly lower or higher expression than either of the parents (see Fig. 2). This large difference in inheritance patterns between maize and *Capsicum* could be due, among other causes, to the fact that maize hybrids result from the cross of two domesticated and highly inbreed lines, while in chili pepper we examined crosses between a wild and a domesticated accessions with high gene expression differences between them—see Figure 1 in^26^.

In maize heterosis has increased yields by at least 15%, and approximately 65% of maize production worldwide is hybrid-based^12^. In contrast, in chili pepper heterosis needs further studies to be extensively employed^20^. In the future, *Capsicum* hybrid vigor adoption could follow the path that happened in sunflower, where heterotic gene pools were developed through the use of wild relatives^14^, in a way similar to the cases of tomato^51^, cotton, sorghum, and others crops which are primarily grown as hybrids^20^.

To fully appreciate heterogeneity in the proportions of the 18 not null sub-models, Fig. 3 presents the percentages of these sub-models per cross, with reference to the total of the null sub-model ($P_1 = F_1 = P_2$). Under homogeneity, each one of the 18 not null sub-models will have an expected frequency of $1/18 \approx 0.0556$ or $\approx 5.56\%$. Nonetheless, there is a very high diversity in the proportions of sub-models; two of them in the intermediate $F_1$ class surpass 10%, while three of them are below 2%—one in class low $F_1$ and two in class high $F_1$, showing that the frequency of inheritance patterns is highly diversified. Also, as shown by the partitions of the bars per cross in Fig. 3, we see how the direction of the cross has a high influence in the inheritance sub-model. However, as mentioned in Results, there is a significant linear correlation with $\hat{r} \approx 0.53$ between the percentages of sub-models at each one of the two crosses, implying that $\approx 28\%;\ (\hat{r}^2)$ of the variation in sub-models is due to the cross of origin.

In^48^ the authors compared gene expression in the parents and reciprocal hybrids of *Arabidopsis* at three different times, finding variations in inheritance patterns during time progression. Even when the availability of genomic resources for important crops has had great progress^52^, the knowledge of the change of gene expression inheritance during fruit development remains poorly studied and understood. Figure 3 shows the dramatic changes that occur in gene expression class along fruit development, without taking into account the null model ($P_1 = F_1 = P_2$). For example, at the mature flower (0 DAA; panels A and B in Fig. 4), the prevalent class in both crosses is “High $F_1$”—and not “Intermediate $F_1$” when all times of fruit development are taken into account (see Fig. 3). In summary, Fig. 3 shows that inheritance class—and also inheritance sub-models (see Supplementary S2), presents a highly dynamic behavior; inheritance pattern change as result of both, time of fruit development and direction of the cross. Because it is unlikely that chili pepper could be exceptional in changing inheritance patterns along fruit development, it is reasonable to assume that such changes will be present in other plant species, even when sub-model frequencies could almost surely be different. This fact is highly relevant for the estimation of eQTLs and, indirectly, for plant breeding programs.

Even when eQTL estimation promises a detailed dissection and understanding of the architecture of gene regulation^53^, our results suggest that time of development is a factor that must be taken into account for this endeavor. For example, in the first large-scale global eQTL study in an *Arabidopsis* population of 211 RILs^54^, the authors report a large and complex set of eQTLs, and show that genetic control of transcript level is highly variable, suggesting that this complexity may be a general characteristic of eukaryotes. However, this study estimated gene expression at a single time of development (6 weeks post-germination), and thus the set of eQTLs obtained does not take into account the additional complexity due to changes in gene expression through plant development. In direct relation with fruit traits, in^55^ the authors studied fruit flesh softening rate in peach, detecting a set of 133 eQTLs related with a strong QTL (LOD of 9.7) affecting the target fruit character. Nevertheless, RNA-Seq sampling in this study was performed at a single time point in fruit development. It appears obvious that gene expression will be changing during peach fruit development, and a RNA-Seq time profile coupled with an inheritance pattern estimation—as the one performed here for chili pepper, could result in a much more detailed and robust understanding of the molecular mechanisms involved in fruit flesh softening.

Our results show that the transcriptome landscape of fruit development dynamically changes through time, presenting the whole spectrum of inheritance patterns in a bidirectional cross between highly contrasting genotypes. We propose that changes in inheritance pattern will be observed in many—if not all, development processes. Therefore, eQTL studies targeting traits related with development will obtain more relevant, detailed and robust conclusions when based into time profiling gene expression experiments.

By performing GO analyses over the 133 sets of genes resulting from combinations of the 7 time points of fruit development with the 19 sub-models of inheritance, we showed that many biological proceses (BP), molecular functions (MF) and cell components (CC) are over represented at particular times of development, having particular inheritance patterns. Even when we are not discussing here the biological relevance of these findings, it is evident that there is a plethora of interesting facts to be explored by researchers interested in particular aspects of this phenomenon.

As mentioned in^39^, omics studies aim to extract relevant messages from large-scale and high-dimensional data sets. To facilitate this goal, we developed an R package containing all results presented here, as well as a function to plot and interpret such results^38^. Coupling this package with the methodology presented in^31^ and the R package *Salsa*^33^, the research community interested in fruit development could fully explore the curated RNA-Seq data from 12 chili pepper accessions, deposited at the NCBI Gene Expression Omnibus^30^ with accession number GSE165448. Data mining of these datasets have already proved to contribute with interesting findings^26,56^.

## Supplementary Information

Supplementary Information.

## Data Availability

The set of RNA-Seq libraries employed in this work have been deposited in NCBI’s Gene Expression Omnibus (GEO)^30^, and are accessible through GEO Series accession number GSE165448 (Link: https://www.ncbi.nlm.nih.gov/geo/query/acc.cgi?acc=GSE165448).
