# Supplementary material for: Inheritance of gene expression throughout fruit development in chili pepper
Source: Sci Rep. 2021 Nov 22;11:22647. doi: 10.1038/s41598-021-02151-z (PMC8609037; doi:10.1038/s41598-021-02151-z)
Supplement: Supplementary file 1 — Supplementary Information. [file 41598_2021_2151_MOESM1_ESM.pdf]

Supplementary file for
"Inheritance of gene expression throughout fruit development in chili pepper"

CHRISTIAN ESCOTO-SANDOVAL, NEFTALÍ OCHOA-ALEJO AND OCTAVIO MARTÍNEZ
COMPUTATIONAL BIOLOGY LAB.
LANGEBIO - CINVESTAV - IRAPUATO

Abstract. Here we present supplements to the methods and results in the main text. Each one of the sections is cited in the main text and here links to the WWW are in **bold face**. Results were processed using R (8) [version 3.4.4], complemented with the R package "*Salsa*" (7), which is fully documented in (4) {Link to package **Salsa**; Link to the paper: "**A method to analyze** ⋯"}, as well as with the R package "*ChiliCross*" (5) {Link to download the package: **ChiliCross**} (See Section S4 here). To reproduce results, as well as to perform further analyses and data-mining you must download and install both packages and load them in your R environment using the commands "`library(Salsa)`" and "`library(ChiliCross)`".

This research was funded by the Consejo Nacional de Ciencia y Tecnología (Conacyt), México; Project Number 1570 and scholarship 630487 to C.E-S.

Contents

S1. Inheritance pattern classification 2
S1.1. Rational of the inheritance pattern classification 2
S1.2. Implementation of statistical tests 3
S2. Tests of independence and consistency in time 5
S2.1. Inheritance patterns depend on cross of origin 5
S2.2. Inheritance patterns within cross depends on time of fruit development 7
S2.3. Relative importance of the cross and time factors on inheritance patterns 12
S2.4. Heat maps of percentages of models as function of time of development 13
S2.5. Consistency of inheritance model through time 16
S3. Gene Ontology (GO) analyses 25
S4. The "*ChiliCross*" R package 30
S4.1. Brief tour into the package 30
S4.2. The `cross.plot()` function 33
References 38

Date: October 18, 2021.

S1. Inheritance pattern classification

In this section we will describe the rational and methods employed to classify the inheritance patterns of each one of the genes by following the schema presented in Fig. 1 in the main text.

S1.1. **Rational of the inheritance pattern classification.** The first step in the description of an inheritance pattern is to assign a "class" and "model", as shown in the main and the blue rectangles of Fig. 1 in the main text. This step is performed by observing –and ranking, the values of the participants in the cross, without doing any statistical tests. Table 1 presents the class and model for each possible observed inheritance case.

Table 1. Class and models for each possible inheritance pattern observed.

Class	m_i	Model
Low F_1	m_1	$F_1 < P_1 < P_2$
	m_2	$F_1 < P_2 < P_1$
Intermediate F_1	m_3	$P_1 < F_1 < P_2$
	m_4	$P_2 < F_1 < P_1$
High F_1	m_5	$P_1 < P_2 < F_1$
	m_6	$P_2 < P_1 < F_1$

In Table 1 we disregard the possibility of ties between the gene expression values in two or three of the participants.

The next step to complete the schema of inheritance classification is to obtain the sub-models, by performing statistical tests for each one of the models observed in Table 1. Table 2 presents the statistical tests and sub-models produced. Such sub-models are presented in each one of the orange rectangles below each one of the models in Fig. 1 of the main text.

Table 2. Sub-models obtained from each model after statistical tests.

		Tests:		Decisions on tests (cells contain resulting sub-models):			
m_i	Model	T_1	T_2	$T_1 \cap T_2$	$T_1 \cap T_2^c$	$T_1^c \cap T_2$	$T_1^c \cap T_2^c$
m_1	$F_1 < P_1 < P_2$	$F_1 \neq P_1$?	$P_1 \neq P_2$?	$F_1 < P_1 < P_2$	$F_1 < P_1 = P_2$	$F_1 = P_1 < P_2$	$F_1 = P_1 = P_2$
m_2	$F_1 < P_2 < P_1$	$F_1 \neq P_2$?	$P_2 \neq P_1$?	$F_1 < P_2 < P_1$	$F_1 < P_2 = P_1$	$F_1 = P_2 < P_1$	$F_1 = P_2 = P_1$
m_3	$P_1 < F_1 < P_2$	$P_1 \neq F_1$?	$F_1 \neq P_2$?	$P_1 < F_1 < P_2$	$P_1 < F_1 = P_2$	$P_1 = F_1 < P_2$	$P_1 = F_1 = P_2$
m_4	$P_2 < F_1 < P_1$	$P_2 \neq F_1$?	$F_1 \neq P_1$?	$P_2 < F_1 < P_1$	$P_2 < F_1 = P_1$	$P_2 = F_1 < P_1$	$P_2 = F_1 = P_1$
m_5	$P_1 < P_2 < F_1$	$P_1 \neq P_2$?	$P_2 \neq F_1$?	$P_1 < P_2 < F_1$	$P_1 < P_2 = F_1$	$P_1 = P_2 < F_1$	$P_1 = P_2 = F_1$
m_6	$P_2 < P_1 < F_1$	$P_2 \neq P_1$?	$P_1 \neq F_1$?	$P_2 < P_1 < F_1$	$P_2 < P_1 = F_1$	$P_2 = P_1 < F_1$	$P_2 = P_1 = F_1$

The rational to classify models into sub-models presented in Table 2 can be confusing at first sight, thus we are going to explain it in detail. Given that models are the observed order (rank) of the expression in three participants, such models include two inequalities; the first inequality between the first and the second participants (left hand side inequality) and the second inequality between the second and the third participants (right hand side inequality). To confirm or reject the first and second inequalities we must perform tests, which are denoted by the questions shown in columns T_1 and T_2, respectively, in Table 2. By answering such two questions (in an affirmative or negative way) we finally obtain the four sub-models presented in columns $T_1 \cap T_2$, $T_1 \cap T_2^c$, $T_1^c \cap T_2$ and $T_1^c \cap T_2^c$ in Table 2.

For example, assume that we observe model m_1, which is $F_1 < P_1 < P_2$ (first row in Table 2). Then T_1 is $F_1 \neq P_1$? while T_2 corresponds to $P_1 \neq P_2$? If both, T_1 and T_2, are answered affirmatively –a result that is shown symbolically as "$T_1 \cap T_2$", then the corresponding sub-model is $F_1 < P_1 < P_2$ –because both inequalities had been confirmed. However, if the first question, $T_1 :\ F_1 \neq P_1$? is responded affirmatively, but the second, $T_2 :\ P_1 \neq P_2$? is responded negatively –a result that is shown symbolically as "$T_1 \cap T_2^c$",

then the corresponding sub-model is $F_1 < P_1 = P_2$. The last two columns, $T_1^c \cap T_2$ and $T_1^c \cap T_2^c$ of the first row in Table 2 complete the logical possibilities; $T_1^c \cap T_2$ means that T_1 was negatively responded while T_2 was affirmatively responded, resulting in sub-model $F_1 = P_1 < P_2$, while when both tests are negatively responded, $T_1^c \cap T_2^c$, the resulting sub-model is $F_1 = P_1 = P_2$.

Table 2 complements the schema given in Fig. 1 of the main text, adding in an explicit way the tests T_1 and T_2 and presenting the sub-models resulting from the four combinations of results in the cells of columns $T_1 \cap T_2$, $T_1 \cap T_2^c$, $T_1^c \cap T_2$ and $T_1^c \cap T_2^c$. It is important to note that all the $6 \times 3 = 18$ sub-models given in the 6 rows of columns $T_1 \cap T_2$, $T_1 \cap T_2^c$ and $T_1^c \cap T_2$ are all different, while the 6 rows in the last column $T_1^c \cap T_2^c$ contain a single sub-model written in different ways, i.e., the "null" sub-model $P_1 = F_1 = P_2$. Thus, in total we have 19 different sub-models, as stated in the main text.

S1.2. **Implementation of statistical tests.** To answer the questions stated in columns T_1 and T_2 in Table 2 we employed the "*edgeR*" R package for empirical analysis of digital gene expression data (10). For each cross, $\mathbf{C}_1$ and $\mathbf{C}_2$, we loaded the raw reads data from our *Salsa* package (7), and performed all pair contrasts shown in Table 3 using the function "`exactTest`" of the package "*edgeR*" (see also Table 1 in main text for the definitions of the crosses).

Table 3. Contrasts between accessions pairs and statistical hypothesis tested.

Row	Accessions	Statistical hypothesis
1	CM *vs.* QU	$\mathcal{H}_0 : P_1 = P_2$ in crosses $\mathbf{C}_1$ and $\mathbf{C}_2$
2	CM *vs.* CQ	$\mathcal{H}_0 : P_1 = F_1$ in cross $\mathbf{C}_1$
3	CM *vs.* QC	$\mathcal{H}_0 : P_1 = F_1$ in cross $\mathbf{C}_2$
4	QU *vs.* CQ	$\mathcal{H}_0 : P_2 = F_1$ in cross $\mathbf{C}_1$
5	QU *vs.* QC	$\mathcal{H}_0 : P_2 = F_1$ in cross $\mathbf{C}_2$

Given that we estimated gene expression at 7 points of chili pepper development: 0, 10, 20, 30, 40, 50 and 60 DAA, the five contrasts shown in Table 3 were performed for each one of the times of development, obtaining a total of $5 \times 6 = 30$ matrices containing the p-values for each gene in each one of the hypothesis tested. Each one of these 30 matrices was transformed from p to q-values by the algorithm in (2), as implemented in the R function "`p.adjust`". With this we were ready to fix an approximate False Discovery Rate (FDR) threshold for all our tests. By considering that in obtaining any of the 19 sub-models presented in Table 2 we will be using the q-values of three of the almost independent contrasts shown in Table 3, we set a minimum q-value of $(0.05)^{1/3}$ at each individual test to obtain an approximate rate of 5% of FDR when comparing any pair of sub-models within the same cross ($\mathbf{C}_1$ or $\mathbf{C}_2$).

By employing this approximate 5% FDR we were able to assign a particular sub-model to each observed model in Table 2, and this assignation was performed for each one of the genes in each one of the two crosses. Then gene expression values for each gene at each one of the participants in the two crosses were standardized, thus these three elements vectors will have an average of one and a standard deviation of one. Standardization allows for an easy comparison between sub-model in genes with discrepant raw expression; however, it is important to remark that statistical analyses to determine sub-models were performed in agreement with the canonical methods, and standardization was performed *a posteriori*, and only as a help for interpretation. All information of the resulting inheritance patterns was gathered into the *ChiliCross* (5) R package into data frames `C1` and `C2` for crosses $\mathbf{C}_1$ and $\mathbf{C}_2$, respectively (See Section S4 for details).

Classification of inheritance patterns into the 19 sub-models presented here is completely general, i.e., it could be applied at any quantitative trait for which the researcher has values in the two parents and at least one of the F_1 generations. The only difference will be in the way in which the tests of significance will be performed. For example, in a case of a quantitative character, as for example plant weight (or any other), the researcher needs individual measurements of the character in each one of the populations P_1, P_2 and F_1 to have a measure of error variance and be able to perform statistical tests, as the ones

shown in Table 3, but employing a suitable statistical test, that will substitute the one employed here for RNA-Seq data *via* the R package *edgeR*.

S2. Tests of independence and consistency in time

Results consist in gene expressions for 22,374 genes in two **reciprocal crosses** which definitions are presented in Table 4, which is also presented in the *ChiliCross* package as data frame "`results.key`".

Table 4. Cross definition

Cross	Definition	P_1	P_2	F_1
$\mathbf{C}_1$	CM ♀ × QU ♂	CM	QU	CQ
$\mathbf{C}_2$	QU ♀ × CM ♂	CM	QU	QC

Table 4 presents the conventions that are followed when denoting each one of the two crosses ($\mathbf{C}_1$ and $\mathbf{C}_2$). The parents of the crosses (P_1 and P_2) are the domesticated accession "CM" (Criollo de Morelos 334) and the wild accession "QU" (Piquín Querétaro). In both cases the F_1 have the same parents, P_1 was CM and P_2 was QU, but the sex of the parents was reversed, i.e., in $\mathbf{C}_1$ the female was CM while pollen donor was QU, and in $\mathbf{C}_2$ the female was QU while pollen donor was CM. The F_1 of each one of the two crosses are denoted with keys "CQ" for $\mathbf{C}_1$ and "QC" for $\mathbf{C}_2$.

Gene expression was estimated throughout the development of the chili pepper fruit at seven time points, say, 0, 10, 20 30, 40, 50 and 60 Days After Anthesis (DAA). These 7 time points include from the mature flower (0 DAA) until the point of full fruit maturation (60 DAA).

To test independence in contingency tables we employed the log likelihood ratio test or "**G-test**" (11) [The G-test was implemented in R by us and it is available upon request (please ask for file `Gtest.R` by email to OM). A large sample alternative to the G-test is the Pearson's "Chi square test", implemented in R in function "`chisq.test()`".].

In this section we demonstrate that inheritance pattern is strongly dependent on the combinations of cross and time of fruit development, evaluate which of these two factors is more important in such determination and study the consistency on time of inheritance patterns.

S2.1. **Inheritance patterns depend on cross of origin.** In the following box we perform **contingency table** analyses to show that inheritance pattern, by class or sub-model, strongly depend on cross of origin, when the time of fruit development is not taken into account, i.e., we will be grouping cases in all 7 times of fruit development.

```
----------------------------------------------------------------------------------------
# Library initialization
> library(Salsa)
> library(ChiliCross)
# Load Gtest functions (optional, you could use chisq.test)
# source("Gtest.R")

# Tabulate the number of cases per class and cross of origin
> temp <- rbind(table(C1$class), table(C2$class))
> attributes(temp)$dimnames[[1]] <- c("C1", "C2")
> temp
   High F1 Intermediate F1 Low F1 null
C1   40857           64823  49248 1690
C2   47116           64240  43512 1750
# NOTE: Here the class "null" includes ONLY cases where the gene was not expressed.
> G.test(temp) # Performs the G-test for independence
             G            df      p.value
 8.042880e+02  3.000000e+00 5.084808e-174
```

```
> chisq.test(temp) # Performs Pearson’s chi square test for independence

Pearson’s Chi-squared test

data:  temp
X-squared = 803.69, df = 3, p-value < 2.2e-16
# Interpretation: The cross of origin strongly influence inheritance class.

# Collapsing data into "Not-null" and "null" columns
> temp2 <- cbind(apply(temp[,1:3], 1, sum), temp[,3])
> attributes(temp2)$dimnames[[2]] <- c("NotNull", "Null")
> temp2
   NotNull  Null
C1  154928 49248
C2  154868 43512
> G.test(temp2)
           G           df      p.value
2.714814e+02 1.000000e+00 5.395787e-61
> chisq.test(temp2)

Pearson’s Chi-squared test with Yates’ continuity correction

data:  temp2
X-squared = 271.19, df = 1, p-value < 2.2e-16
# Interpretation: The number of expressed genes is different by cross.

# Now test by criteria cross and sub-model
# Note that here sub-model P1=F1=P2 could result from two sources:
# 1 - Genes that are not expressed in the cross participants or
# 2 - Genes with not significant differences (at 5% of FDR).
> temp <- cbind(table(C1$sub.model), table(C2$sub.model))
> attributes(temp)$dimnames[[2]] <- c("C1", "C2")
> head(temp)
           C1   C2
F1<P1<P2  712 3911
F1<P1=P2 2116 5840
F1<P2<P1  601  968
F1<P2=P1 2272 2746
F1=P1<P2 3622  234
F1=P2<P1 2472 2705
> G.test(temp)
       G       df  p.value
12816.68    18.00     0.00
> chisq.test(temp)

Pearson’s Chi-squared test

data:  temp
X-squared = 11768, df = 18, p-value < 2.2e-16

# Now by grouping sub-model in "P1=F1=P2" and "others"
> temp[attributes(temp)$dimnames[[1]]=="P1=F1=P2",]
```

```
    C1     C2
114436 100474
> apply(temp[attributes(temp)$dimnames[[1]]!="P1=F1=P2",],2,sum)
   C1    C2
42182 54394

> temp2 <- rbind(temp[attributes(temp)$dimnames[[1]]=="P1=F1=P2",],
     apply(temp[attributes(temp)$dimnames[[1]]!="P1=F1=P2",],2,sum))
> attributes(temp2)$dimnames[[1]] <- c("P1=F1=P2", "other")
> temp2
             C1     C2
P1=F1=P2 114436 100474
other     42182  54394
> G.test(temp2)
       G       df  p.value
2446.217    1.000    0.000
> chisq.test(temp2)

Pearson's Chi-squared test with Yates' continuity correction

data:  temp2
X-squared = 2441.1, df = 1, p-value < 2.2e-16
```

Results presented in the previous box show that inheritance patterns without taking into account the time of fruit development, and classified by either class or sub-model, strongly depends on the cross of origin.

S2.2. **Inheritance patterns within cross depends on time of fruit development.** For each cross ($\mathbf{C}_1$ and $\mathbf{C}_2$) we have seven times of fruit development. The following box presents analyses that show that both, class and sub-model within each cross strongly depend on the time of fruit development.

```
# Analyses of contingency for cross C1
# Class within C1 by time
> table(C1$class, C1$time)

                     00    10    20    30    40    50    60
  High F1         12976  4640  4948  6658  3035  3011  5589
  Intermediate F1  6003 12841 11403  9956  6190  8833  9597
  Low F1           3296  4698  5891  5546 12971 10221  6625
  null               99   195   132   214   178   309   563

> round(expected(table(C1$class, C1$time))) # Expected under independence
                  00   10   20   30   40   50   60
High F1         5837 5837 5837 5837 5837 5837 5837
Intermediate F1 9260 9260 9260 9260 9260 9260 9260
Low F1          7035 7035 7035 7035 7035 7035 7035
null             241  241  241  241  241  241  241

> G.test(table(C1$class, C1$time))
       G       df  p.value
24237.95    18.00     0.00
```

```
> chisq.test(table(C1$class, C1$time))

Pearson's Chi-squared test

data:  table(C1$class, C1$time)
X-squared = 26430, df = 18, p-value < 2.2e-16

# Sub-model  within C1 by time
> table(C1$sub.model, C1$time)

               00    10    20    30    40    50    60
  F1<P1<P2    279     7     7    35    10   368     6
  F1<P1=P2    492    11    18   141   360  1077    17
  F1<P2<P1    209    13    19   139    16   203     2
  F1<P2=P1    359    29    58   202  1010   603    11
  F1=P1<P2    205   759   273   292   284  1611   198
  F1=P2<P1    169   137   311   751    66   862   176
  P1<F1<P2    191   324   341   551    94   877    11
  P1<F1=P2    647   177   482  1605    80  1151   150
  P1<P2<F1     44    13     9   168    17   159     0
  P1<P2=F1    738   153   295  1215    26   492   323
  P1=F1<P2    390  1586   841   562  1039  1550    58
  P1=F1=P2  14250 16003 18201 14549 18478 11760 21195
  P1=P2<F1    257    52    80   245   146   154     7
  P2<F1<P1    116   137   195   321    68   210     8
  P2<F1=P1    899  2167   619   427   383   177    96
  P2<P1<F1    156     4    12    29     6    59     1
  P2<P1=F1   2346   706   260   191    90   123    71
  P2=F1<P1    127    91   337   823   125   765    39
  P2=P1<F1    500     5    16   128    76   173     5
> round(expected(table(C1$sub.model, C1$time))) # Expected under independence
             00    10    20    30    40    50    60
F1<P1<P2    102   102   102   102   102   102   102
F1<P1=P2    302   302   302   302   302   302   302
F1<P2<P1     86    86    86    86    86    86    86
F1<P2=P1    325   325   325   325   325   325   325
F1=P1<P2    517   517   517   517   517   517   517
F1=P2<P1    353   353   353   353   353   353   353
P1<F1<P2    341   341   341   341   341   341   341
P1<F1=P2    613   613   613   613   613   613   613
P1<P2<F1     59    59    59    59    59    59    59
P1<P2=F1    463   463   463   463   463   463   463
P1=F1<P2    861   861   861   861   861   861   861
P1=F1=P2  16348 16348 16348 16348 16348 16348 16348
P1=P2<F1    134   134   134   134   134   134   134
P2<F1<P1    151   151   151   151   151   151   151
P2<F1=P1    681   681   681   681   681   681   681
P2<P1<F1     38    38    38    38    38    38    38
P2<P1=F1    541   541   541   541   541   541   541
P2=F1<P1    330   330   330   330   330   330   330
P2=P1<F1    129   129   129   129   129   129   129
> G.test(table(C1$sub.model, C1$time))
```

```
       G       df  p.value
38962.96   108.00     0.00
> chisq.test(table(C1$sub.model, C1$time))

Pearson's Chi-squared test

data:  table(C1$sub.model, C1$time)
X-squared = 42564, df = 108, p-value < 2.2e-16

# Analyses of contingency for cross C2
# Class within C2 by time
> table(C2$class, C2$time)

                     00    10    20    30    40    50    60
  High F1         12395  6067  5714  6509  5003  4153  7275
  Intermediate F1  6901 10579  7156  9267  8961 11517  9859
  Low F1           2996  5527  9365  6376  8208  6340  4700
  null               82   201   139   222   202   364   540
> round(expected(table(C2$class, C2$time))) # Expected under independence
                  00   10   20   30   40   50   60
High F1         6731 6731 6731 6731 6731 6731 6731
Intermediate F1 9177 9177 9177 9177 9177 9177 9177
Low F1          6216 6216 6216 6216 6216 6216 6216
null             250  250  250  250  250  250  250
> G.test(table(C2$class, C2$time))
       G       df  p.value
12678.77    18.00     0.00
> # Sub-model  within C1 by time
> table(C2$sub.model, C2$time)

              00    10    20    30    40    50    60
  F1<P1<P2   505   748  1165   391   133   829   140
  F1<P1=P2   709   277  2518   899   702   424   311
  F1<P2<P1    55    96   396   362     2    47    10
  F1<P2=P1   279    87  1584   477   103   147    69
  F1=P1<P2     0   119     2     2     3   106     2
  F1=P2<P1   156   717   485   752    58   431   106
  P1<F1<P2   484   297   363   700    37   916   122
  P1<F1=P2  1164   412   438  1561   161  2077   645
  P1<P2<F1   116    93   300   315     3    86    13
  P1<P2=F1   655   550   326  1178    88   566   231
  P1=F1<P2   447   775  1322   729   231  1017   315
  P1=F1=P2 10449 15359  9865 12221 20230 13319 19031
  P1=P2<F1   879   222   849   493    40   121    61
  P2<F1<P1   489   198   381   414    19   241    67
  P2<F1=P1  1229  1085   710   420   231   257   347
  P2<P1<F1   689    24   159    70     0    45     5
  P2<P1=F1  1450   692   375   173    63   246   125
  P2=F1<P1   400   338   493   780    61   931   183
  P2=P1<F1  2137    84   504   215     7   204    51
> round(expected(table(C2$sub.model, C2$time))) # Expected under independence
           00    10    20    30    40    50    60
```

```
F1<P1<P2   563   560   562   559   560   556   551
F1<P1=P2   841   836   838   835   836   830   823
F1<P2<P1   139   139   139   138   139   138   136
F1<P2=P1   395   393   394   393   393   390   387
F1=P1<P2    34    34    34    33    34    33    33
F1=P2<P1   389   387   388   387   387   384   381
P1<F1<P2   420   418   419   418   418   415   412
P1<F1=P2   930   925   927   924   925   918   910
P1<P2<F1   133   133   133   132   133   132   131
P1<P2=F1   517   515   516   514   515   511   507
P1=F1<P2   696   692   694   692   692   687   682
P1=F1=P2 14462 14385 14425 14372 14385 14279 14165
P1=P2<F1   384   382   383   381   382   379   376
P2<F1<P1   260   259   260   259   259   257   255
P2<F1=P1   616   613   614   612   613   608   603
P2<P1<F1   143   142   142   142   142   141   140
P2<P1=F1   450   447   449   447   447   444   440
P2=F1<P1   459   456   457   456   456   453   449
P2=P1<F1   461   458   460   458   458   455   451
> G.test(table(C2$sub.model, C2$time))
       G       df  p.value
42727.46   108.00     0.00
> chisq.test(table(C2$sub.model, C2$time))

Pearson's Chi-squared test

data:  table(C2$sub.model, C2$time)
X-squared = 47431, df = 108, p-value < 2.2e-16
-----------------------------------------------------------------------------------------
```

From the results presented above we see that inheritance patterns within cross, when classified in classes as well as in sub-models, strongly depend on time of fruit development in both crosses.

An interesting question is how different are the numbers of sub-models per time that appear in each one of the the two crosses. This question can be investigated by performing an individual **G-test** (11) for each one of the $19 \times 7 = 133$ combinations of sub-models $\times$ time of development that were estimated in the tables of the two crosses, $\mathbf{C}_1$ and $\mathbf{C}_2$. The null hypothesis in those tests is that the frequency of sub-models in a time of development is equal in the two crosses, *vs.* the alternative of such numbers being different. Because we are going to be performing 133 hypothesis tests, we will apply the **Bonferroni correction** to achieve a small Type I error. Also, because we want to have a high confidence in that the rejection, we will select a very small probability of Type I error, say $\alpha = 1 \times 10^{-5} = 0.00001$ or 0.001%. Thus, the threshold of significance in each one of the individual tests will be given by $\alpha^* = 0.00001/133 \approx 7.52 \times 10^{-8}$. Next box presents the R calculations performed to conclude in which cases the numbers of sub-models differ between crosses.

```
-----------------------------------------------------------------------------------------
> # Observed frequencies in the C1 and C2 tables.
> temp.C1 <- table(C1$sub.model, C1$time)
> temp.C2 <- table(C2$sub.model, C2$time)
> # Expected frequencies of cells if the two tables are equal
> temp.C1.C2 <- (temp.C1+temp.C2)/2
> # Individual G terms for each cross
> temp.G.C1 <- (temp.C1+1)*log((temp.C1+1)/temp.C1.C2)
```

```
> temp.G.C2 <- (temp.C2+1)*log((temp.C2+1)/temp.C1.C2)
> # Value of the G statistic for each one of the 133 tests
> temp.G <- temp.G.C1+temp.G.C2
> # These values will be approximately distributes as a Chi square with 1 df.
> # Threshold for the G value to be considered as significant
> temp.T <- qchisq(0.00001/133, df=1, ncp = 0, lower.tail = F)
> temp.T
[1] 28.92623
> # Now we obtain a matrix where 1 means a significant difference
> # while 0 means a not significant difference (alpha corrected = 0.00001/133)
> temp.sigG <- 1*(temp.G >= temp.T)
# Now annotate that matrix with the total of significant
# rows and columns
> temp.sigG <- cbind(temp.sigG, apply(temp.sigG, 1, sum))
> temp.sigG <- rbind(temp.sigG, apply(temp.sigG, 2, sum))
> attributes(temp.sigG)$dimnames[[1]][20] <- "TotTime"
> attributes(temp.sigG)$dimnames[[2]][8] <- "TotSubMod"
> temp.sigG
         00 10 20 30 40 50 60 TotSubMod
F1<P1<P2  1  1  1  1  1  1  1         7
F1<P1=P2  0  1  1  1  1  1  1         6
F1<P2<P1  1  1  1  1  0  1  0         5
F1<P2=P1  0  0  1  1  1  1  0         4
F1=P1<P2  1  1  1  1  1  1  1         7
F1=P2<P1  0  1  0  0  0  1  0         2
P1<F1<P2  1  0  0  0  0  0  1         2
P1<F1=P2  1  1  0  0  0  1  1         4
P1<P2<F1  0  1  1  0  0  0  0         2
P1<P2=F1  0  1  0  0  0  0  0         1
P1=F1<P2  0  1  1  0  1  1  1         5
P1=F1=P2  1  0  1  1  1  1  1         6
P1=P2<F1  1  1  1  1  1  0  0         5
P2<F1<P1  1  0  1  0  0  0  0         2
P2<F1=P1  0  1  0  0  0  0  1         2
P2<P1<F1  1  0  1  0  0  0  0         2
P2<P1=F1  1  0  0  0  0  0  0         1
P2=F1<P1  1  1  0  0  0  0  1         3
P2=P1<F1  1  1  1  0  1  0  0         4
TotTime  12 13 12  7  8  9  9        70

# Percentage of significant differences per time
> round(100*temp.sigG[20,]/c(rep(19,7), 133),2)
       00        10        20        30        40        50        60
    63.16     68.42     63.16     36.84     42.11     47.37     47.37
TotSubMod
    52.63
# Percentage of significant differences per sub-model
> round(100*temp.sigG[,8]/c(rep(7,19), 133),2)
F1<P1<P2 F1<P1=P2 F1<P2<P1 F1<P2=P1 F1=P1<P2 F1=P2<P1 P1<F1<P2 P1<F1=P2
  100.00    85.71    71.43    57.14   100.00    28.57    28.57    57.14
P1<P2<F1 P1<P2=F1 P1=F1<P2 P1=F1=P2 P1=P2<F1 P2<F1<P1 P2<F1=P1 P2<P1<F1
   28.57    14.29    71.43    85.71    71.43    28.57    28.57    28.57
```

```
P2<P1=F1 P2=F1<P1 P2=P1<F1  TotTime
   14.29    42.86    57.14    52.63
-------------------------------------------------------------------------------------
```

From the previous results we conclude that in the majority of the cases, 70/133 or ≈ 52.63%, there is a highly significant (0.001%) difference between the numbers of sub-models per cross. Thus, there is a strong effect of the direction of the cross in the inheritance patterns. The more alike time of development between crosses, with only 7/19; ≈ 36.84% of significant differences is 30 DAA, while the sub-model with less significant differences between crosses is $P_1 < P_2 = F_1$ with only 1/7; ≈ 14.29%. In contrast, sub-models $F_1 < P_1 < P_2$ and $F_1 = P_1 < P_2$ presented differences in all times of development.

S2.3. **Relative importance of the cross and time factors on inheritance patterns.** Previously we have seen that both, time of development and cross of origin affect the inheritance pattern, i.e., the sub-model detected in the genes. Here we investigated the relative importance of those two factors.

First, by fitting generalized linear models under the Poisson link we corroborated the results of the previous section, i.e., that the cross of origin as well as the time of development have influence in the inheritance pattern (results not shown). However, to obtain an approximate ratio of the importance of cross / time of development we used ANOVA for the rank of the number of models as function of those factors. Next box presents the R calculations performed.

```
-------------------------------------------------------------------------------------
# First obtain a data.frame with the number of sub-models for each combination
# of cross and time of development.
> temp <- expand.grid(c('C1', 'C2'), unique(C1$time), c(0:18), stringsAsFactors=F)
> names(temp) <- c('cross', 'time', 'num.sub.mod')
> temp <- temp[,c(3,1,2)]
> temp <- temp[order(temp$num.sub.mod, temp$cross, temp$time),]
> attributes(temp)$row.names <- c(1:266)
> temp$sub.model <- ''
# Give explicit value to sub-model
for(i in 1:19){
temp$sub.model[temp$num.sub.mod==i-1] <- all.models$sub.model[i]
}
> temp <- temp[,c(1,4,2,3)]
> names(temp)[1] <- 'nsm' # Number of sub-models
# Fill nsm:
for(i in 1:266){
if(temp$cross[i]=='C1'){
temp$nsm[i] <- nrow(C1[(C1$sub.model==temp$sub.model[i])&(C1$time==temp$time[i]),])
} else {
temp$nsm[i] <- nrow(C2[(C2$sub.model==temp$sub.model[i])&(C2$time==temp$time[i]),])
}
}
> temp$r.nsm <- rank(temp$nsm) # Rank of number of sub-models

# We have a factorial design with 19 "replicates" per combination
# of Cross X Time of development
> table(temp$cross, temp$time)
     00 10 20 30 40 50 60
  C1 19 19 19 19 19 19 19
  C2 19 19 19 19 19 19 19
```

```
# Perform the analysis of variance
> temp.aov <- aov(r.nsm ~ cross*time, data=temp)
> summary(temp.aov)
             Df  Sum Sq Mean Sq F value   Pr(>F)
cross         1  326060  326060  79.684  < 2e-16 ***
time          6  130988   21831   5.335 3.34e-05 ***
cross:time    6   80178   13363   3.266  0.00413 **
Residuals   252 1031158    4092
---
Signif. codes:  0 ?***? 0.001 ?**? 0.01 ?*? 0.05 ?.? 0.1 ? ? 1

> plot(temp.aov) # To check ANOVA assumptions (plots not shown)
> hist(temp.aov$residuals) # Check distribution of residuals (not shown)

# Conclusion: Both, cross and time are important in determining
# the rank of the number of models.
# Quantitative comparisons of the standard deviation of the mean squares can be done:
> due2cross <- sqrt(326060)
> due2time <- sqrt(21831)
> due2cross
[1] 571.0166
> due2time
[1] 147.7532
> due2cross/due2time
[1] 3.864666
```

The rank of the number of sub-models in each combination of cross × time of development reflects how close is the number of sub-models in each one of those combinations and thus the ANOVA with those factors can be used to evaluate the relative importance of them. Checking the residuals of the ANOVA does not show strong departures from ANOVA assumptions and a histogram of the residuals shows a bell shaped curve, thus we can consider this analysis as valid. From this analysis we conclude that cross of origin is approximately 3.8 times more important that time of fruit development to determine de rank of the number of sub-models obtained.

S2.4. **Heat maps of percentages of models as function of time of development.** For a better understanding of inheritance patterns during the development of the fruit we present heat maps of the percentages of the models (see blue rectangles in Fig. 1 in main text) as well as the null model, $P_1 = F_1 = P_2$, but in this case only for genes had an estimated expression of zero (non expressed genes). The plots were obtained with the R package "*pheatmap*" (6), and, for brevity, R calculations are not shown.

Figures 1, 2 and 3 present heat maps for the percentage per time of development of each one of the models mentioned above for crosses $\mathbf{C}_1$, $\mathbf{C}_2$ and taking both crosses into account, respectively. In all three figures the dendrograms grouping patterns (left hand side margin) as well as times of development (upper margin) were obtained using Euclidean distance and the "average" (UPGMA) hierarchical algorithm on the percentages of patterns.

In Figure 1 we can observe the heterogeneity in the percentages of expression patterns per time of development in cross $\mathbf{C}_1$. Variation in these percentages goes from less than 1% up to 38% with a mean of 14%. The lowest percentages are for non-expressed genes ($P_1 = F_1 = P2$) in the first row of the matrix in Figure 1, and that expression pattern is segregated as the one farther away in the dendrogram of patterns (left hand side margin). In that dendrogram patterns with lowest expression in the F_1, say,

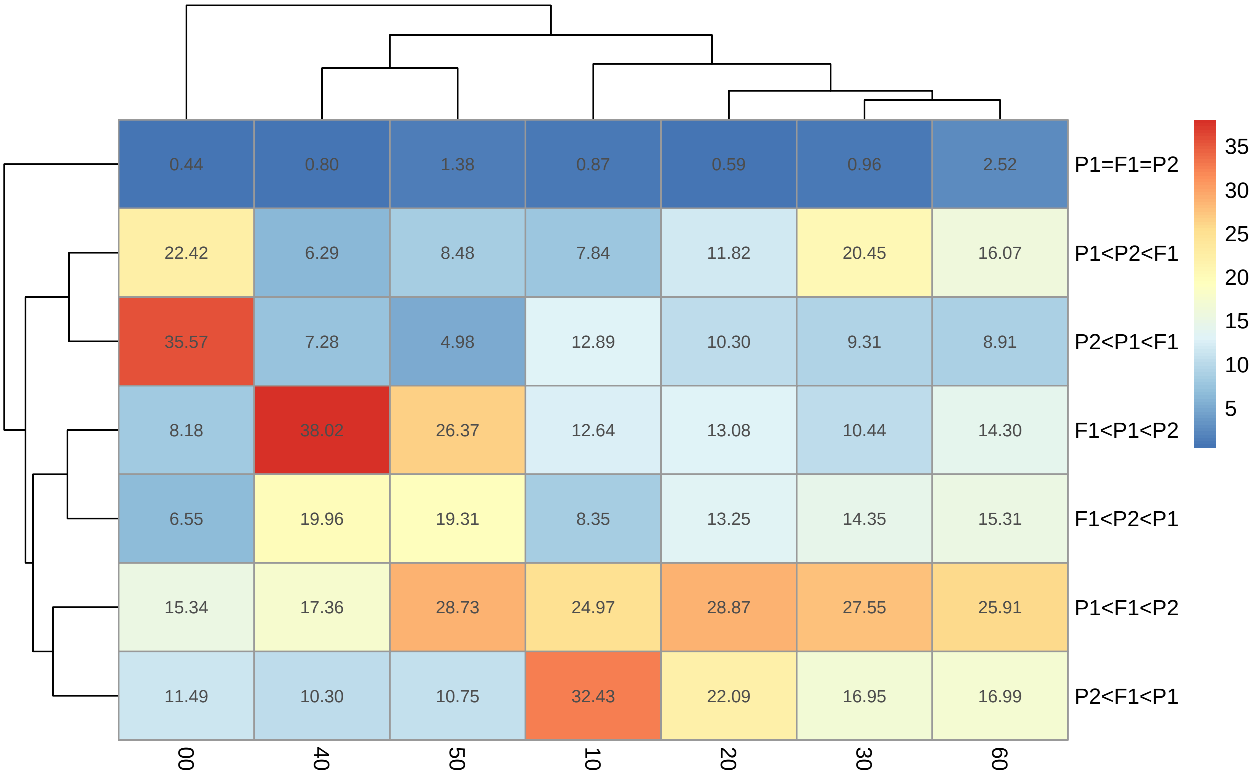

FIGURE 1. Heat map for percentages of models (Y-axis) in $\mathbf{C}_1$ in the different times (X-axis). Percentages of each one of the times add to 100%.

$F_1 < P_1 < P2$ and $F_1 < P_2 < P1$, are grouped together at the lowest Euclidean distance (rows 4 and 5 in the matrix). Approximately at the same low Euclidean distance are formed the groups with patterns $\{P_1 < P_2 < F1\}, \{P_2 < P_1 < F_1\}$, which have as common factor to present the lowest expression in the F_1, and also the groups with patterns $\{P_1 < F_1 < P_2\}, \{P_2 < F_1 < P_1\}$, which have as common factor to show the values for the F_1 in the middle, i.e., with intermediate inheritance. On the other hand, the dendrogram grouping times of development in the upper margin of the figure, shows that the expression times closest in percentages of patterns are times 30 and 60 DAA, while the time at 0 DAA (mature flower) is the one that is farther away from the other times of development.

Figure 2 also shows the heterogeneity in the percentages of expression patterns per time of development in cross $\mathbf{C}_2$. As in Figure 1 –for $\mathbf{C}_1$, the percentages per time present an ample range, from a minimum of less than 1% up to a maximum of 36%, with a mean of 14%, and the pattern $P_1 = F_1 = P2$ (first row of the matrix in Figure 2) is segregated in the left hand side dendrogram by being the case with lowest pattern percentages. The more alike patterns by percentages of cases in this figure are patterns $F_1 < P_1 < P_2$ and $F_1 < P_2 < P_1$, which have in common the fact of having the lowest expression in the F_1. Apart from pattern $P_1 = F_1 = P2$, the pattern $P_1 < F_1 < P2$ –which overall is the most frequent, is segregated as a single branch in the dendrogram of patterns. The dendrogram grouping times of development in the upper margin of the figure, segregates the mature flower (0 DAA) as a single branch (first column of the matrix), and this case is shared with Figure 1. Also as in Figure 1 times 30 and 60 are linked in a cluster by sharing alike percentages of patterns, but otherwise figures 1 and 2 present enough differences to conclude that the percentages of inheritance patterns between the two crosses are different, and this in turn implies that the direction of the cross produces high differences in the landscape of the transcriptomes.

Figure 3 presents the percentages of patterns per time of development obtained by adding the values obtained in each cross, i.e., without distinguishing by cross. This figure share with figures 1 and 2 the

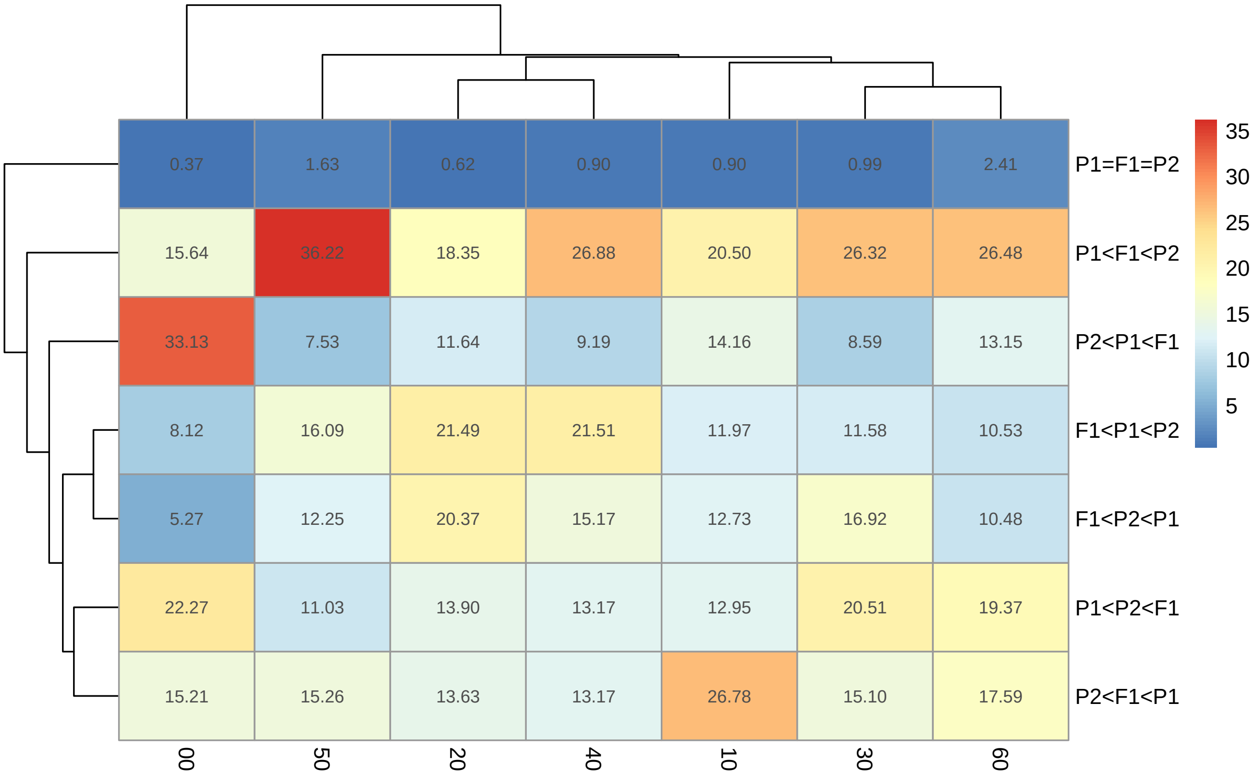

FIGURE 2. Heat map for percentages of models (Y-axis) in $\mathbf{C}_2$ in the different times (X-axis). Percentages of each one of the times add to 100%.

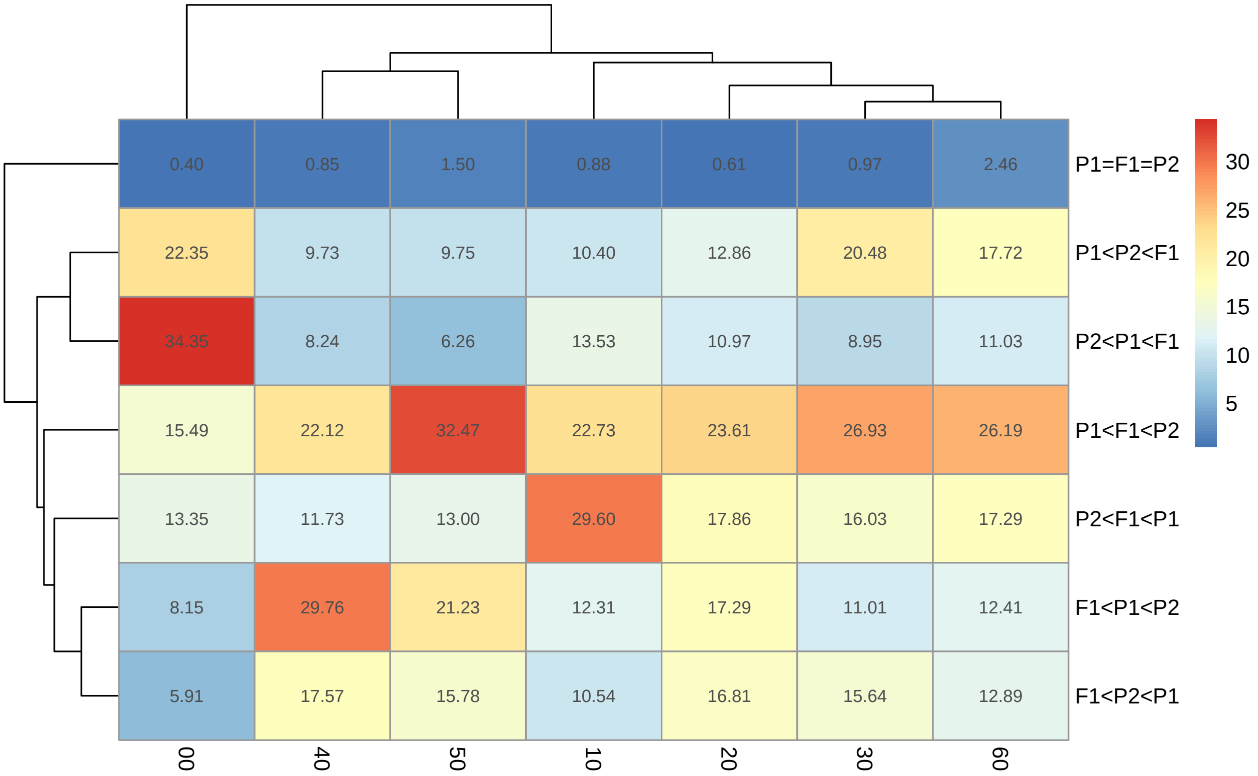

FIGURE 3. Heat map for percentages of models (Y-axis) in both crosses ($\mathbf{C}_1$ and $\mathbf{C}_2$) in the different times (X-axis). Percentages of each one of the times add to 100%.

facts that pattern $P_1 = F_1 = P_2$ and time 0 DAA (mature flower) are segregated as single branches in the corresponding dendrograms. Also, patterns where the F_1 have the lowest values (rows 6 and 7 in the matrix) show the more alike percentages of expression patterns, and this topological characteristic is shared by figures 1 and 2. Patterns having the highest values in the F_1 (rows 2 and 3 in the matrix) share an alike profile of percentages, and thus are grouped at small Euclidean distance in the dendrogram of the left hand side margin in Figure 3.

As a general conclusion derived from the heat maps presented in Figures 1, 2 and 3 we can say that even when the transcriptome landscape of the crosses is affected by the direction of the cross, there is congruence between the percentages of patterns expressed during fruit development.

S2.5. **Consistency of inheritance model through time.** We will say that the inheritance of the expression model of a gene is consistent between the two crosses if that model is identical in crosses $\mathbf{C}_1$ and $\mathbf{C}_2$. See the definition of gene models m_1 to m_6 in Fig. 1 on the main text. Here, as in the previous sections, model $P_1 = F_1 = P_2$ is taken in strict sense as genes with no expression detected. We have a total of 22,374 genes, which were evaluated at 7 different times of fruit development –from 0 to 60 DAA, sampling every 10 days, thus we have a total of $22,374 \times 7 = 156,618$ inheritance models to be evaluated. To evaluate the consistency of inheritance of these sets of genes, we measured the proportional size of the intersection of genes having the same model (consistent genes) with reference to the size of the union of such groups. Let's denote as S_{ij1}, S_{ij2} the set of genes with a pattern i; $i = 1, 2, \cdots, 7$ at time j; $j = 0, 10, \cdots, 60$ DAA in crosses $\mathbf{C}_1$ and $\mathbf{C}_2$, respectively. Then, the consistency between the two sets, S_{ij1} and S_{ij2}, is measured by

$$c_{ij} = \frac{|S_{ij1} \cap S_{ij2}|}{|S_{ij1} \cup S_{ij2}|} \tag{1}$$

i.e., c_{ij} is the quotient of the size of the intersection of both sets divided by the size of their union. Clearly $0 \leq c_{ij} \leq 1$, and the value $c_{ij} = 0$ happens when the two sets are disjoint, having an empty intersection, while the value $c_{ij} = 1$ is reach only when the sets are identical.

To be able to compare the numbers of observed consistent genes, say the vector $f_{ij} = |S_{ij1} \cap S_{ij2}|$; $i = 1, 2, \cdots, 7$, $j = 0, 10, \cdots, 60$, we need to calculate the expected number for each one of those numbers under the hypothesis of independence. First, consider the estimated probabilities of obtaining each number of patterns, say $|S_{ij1}|$, $|S_{ij2}|$ in crosses $\mathbf{C}_1$ and $\mathbf{C}_2$, respectively. Those probabilities are estimated by $\hat{p}_{ij1} = |S_{ij1}|/\sum_{ij}|S_{ij1}|$ and $\hat{p}_{ij2} = |S_{ij2}|/\sum_{ij}|S_{ij2}|$, and note that $\sum_{i,j}|S_{ij1}| = \sum_{i,j}|S_{ij2}| = 156618$ –the total number of genes $\times$ the seven times estimated. Thus, under the hypothesis of independence we have that the expected value of each f_{ij} is given by $E[f_{ij}] = \hat{p}_{ij1}\hat{p}_{ij2}\sum_{ij}|S_{ij1}|$, where the hypothesis of independence is used to multiply the estimated probabilities $\hat{p}_{ij1}$ and $\hat{p}_{ij2}$, while the sum of all models is, indistinctly equal to $\sum_{i,j}|S_{ij1}|$ or $\sum_{i,j}|S_{ij2}|$ (because those two quantities are equal). Table 5 presents the main statistics for the distributions of the 49 values of f_{ij} and $E[f_{ij}]$.

Table 5. Comparison of the main statistics for the distributions of the observed (f_{ij}) and expected ($E[f_{ij}]$) numbers of consistent patterns (rounded values).

	Min.	1st Qu.	Median	Mean	3rd Qu.	Max.	$\sum_{ij}$
f_{ij}	68	995	1492	1858	2328	6201	91046
$E[f_{ij}]$	0	26	53	86	99	377	4229

In Table 5 we can appreciate how the distributions of the values of the observed (f_{ij}) and expected ($E[f_{ij}]$) numbers of consistent genes are completely different. For example, the mean of the number of observed values, f_{ij}, is 1,858, while the mean of the expected values, $E[f_{ij}]$, is of only 86, a ratio of $1,858/86 \approx 21.6$ times larger number of observed compared with expected consistent genes. In the same sense, the total sum of observed consistent genes, $\sum_{ij} f_{ij} = 91,046$, is approximately 21.6 times larger than the sum of the expected consistent genes under the hypothesis of independence, $\sum_{ij} E[f_{ij}] = 4,229$

(91, 046/4, 229 ≈ 21.6). Note that both, observed and expected number of consistent genes, depends on the fully independently estimated expression values of the F_1's, CQ and QC for crosses $\mathbf{C}_1$ and $\mathbf{C}_2$, respectively. From this analysis we can conclude that the number of observed consistent genes, i.e., genes with the same expression pattern in both crosses, is at least 21 times more abundant that the number of consistent genes expected under independence.

The $7 \times 7 = 49$ consistence values, c_{ij}, varied from a minimum of ≈ 16% up to a maximum of ≈ 72%, having a mean value of ≈ 43% and a standard deviation of ≈ 14%. Table 6 presents the statistics (minimum, mean, maximum and standard deviation S) for the values of consistency, c_i, for each one of the 7 patterns (variation among the 7 times).

TABLE 6. Statistics for c_i (consistence in % per pattern over all 7 times).

	$P_1 = F_1 = P_2$	$P_1 < F_1 < P_2$	$P_1 < P_2 < F_1$	$F_1 < P_1 < P_2$	$F_1 < P_2 < P_1$	$P_2 < P_1 < F_1$	$P_2 < F_1 < P_1$
Min.	60	30	16	30	27	22	33
Mean	67	46	34	37	39	35	41
Max.	72	53	61	50	46	68	55
S	4	8	15	6	7	16	8

In Table 6 we can see that the highest consistency is present for the pattern $P_1 = F_1 = P_2$ (first column), where the mean consistency was ≈ 67%, i.e., around 67% of the genes with this pattern are shared among all times. On the other hand, the pattern with less consistent genes is $P_1 < P_2 < F_1$ with a mean consistence among times of ≈ 34%. Table 7 presents the statistics (minimum, mean, maximum and S) for the values of consistency, c_j, for each one of the 7 times (variation among the 7 patterns).

TABLE 7. Statistics for c_j (consistence in % per time over all 7 patterns).

	0	10	20	30	40	50	60
Min.	33	27	24	37	16	25	34
Mean	52	42	36	47	35	42	44
Max.	68	64	66	70	65	70	72
S	12	15	14	11	16	16	14

In Table 7 we can see that the highest consistency is present for the time "00" i.e., 0 DAA (first column), where the mean consistency was ≈ 52%. On the other hand, the time with less consistent genes is 40 DAA, with a mean consistence among patterns of ≈ 35%. Comparing Tables 6 and 7, we can notice that the standard deviation (S) of the consistency among times in Table 7 is in general larger than the standard deviation of the consistency between patterns, presented in Table 6. This means that, in general, there is more variation in consistency among times than among patterns. However, for a better understanding of the consistency at each pair of sets, c_{ij}, Figure 4 presents a heat map of the full consistency matrix.

In Figure 4 we can observe with detail the heterogeneity of consistencies, c_{ij}, which vary between the minimum of ≈ 16% up to a maximum of ≈ 72%, with a mean of ≈ 43%. The dendrogram for patterns, in the left hand side, shows that the most consistent pattern is $P_1 = F_1 = P_2$ (first row of the matrix) and it is well segregated from the other patterns as a single external branch. The topology of this dendrogram is identical to the topology of the dendrogram for patterns presented in Figure 1 for the percentages of patterns in $\mathbf{C}_1$. As in that dendrogram, the patterns of the left hand side dendrogram in Figure 4 form groups $g_1 = \{P_1 < P_2 < F1, P_2 < P_1 < F_1\}$ (rows 3 and 4 in the matrix), $g_2 = \{F_1 < P_1 < P2, F1 < P_2 < P_1\}$ (rows 5 and 6 in the matrix) and $g_3 = \{P_1 < F_1 < P_2, P_2 < F_1 < P_1\}$ (rows 6 and 7 in the matrix). g_1 has as common feature the fact that the F_1's represent the largest values of the patterns, while in opposition in g_2 the F_1's are the smallest values in the patterns and, finally, in g_3 the F_1's are intermediate between the two parents. This implies that closeness in consistency is governed mainly by the positions of the F_1's values. On the other hand, the dendrogram grouping times of expression, at the upper margin of the figure, shows that the column of consistencies for time 0 (first column of the matrix)

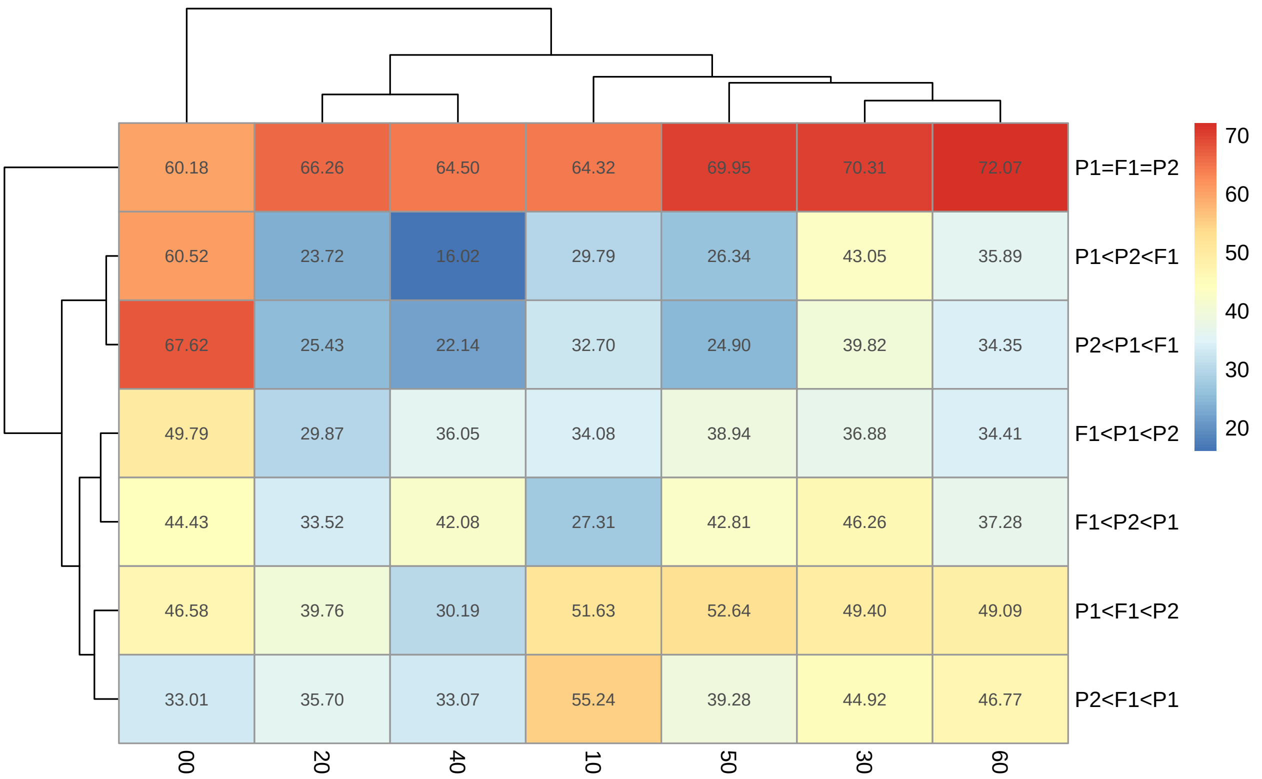

FIGURE 4. Heat map for percentages of consistency, c_{ij}, between sets formed by genes with the different patterns (Y-axis) at each one of the different times (X-axis).

is a single branch of the dendrogram, well segregated from all other vectors of consistency per time. This fact can also be observed in the corresponding dendrograms in Figures 1, 2 and 4, which present heat maps for the proportions of patterns happening in $\mathbf{C}_1$, $\mathbf{C}_2$ and both crosses, respectively. From this we can conclude that the transcriptome landscape in the mature flower (time 0 DAA of development) is the most particular one of all times sampled.

Up to this point we have a detailed panorama of the inheritance patterns of the genes at each one of the times sampled. We conclude that, in average overall times, the most frequent inheritance pattern is $P_1 < F_1 < P_2$ —which can also be written as "CM $< F_1 <$ QU". This pattern comprises $\approx 24\%$ of all cases, and means that the domesticated parent frequently ($\approx 24\%$) presents an standardized expression lower than the one in the wild parent, while the value of the F_1 is intermediate to the expression of the parents (see Also panel (B) in Fig. 2 of the main text). Also, by examining the percentages of pattern $P_1 < F_1 < P_2$ segregating by time in both crosses (Figure 3, row 4 in the matrix), such percentages have high values in all times > 0 DAA, and also show a high consistence in both crosses (row six in Figure 4).

Another question of interest is if, for a given gene, inheritance pattern is relatively consistent through the time of fruit development. We sampled 7 times: $0, 10, \cdots, 60$ DAA, and we have that at each time there could be 7 different inheritance patterns (models), thus we could have a total of $7^7 = 823,543$ different inheritance pattern profiles during the sampled times of fruit development. Here we use the acronym "IPP" for "**Inheritance Pattern Profile**" through time of development. This number of IPP, 823,543, is very large, considering that we are studying a total of only 2,2374 genes. If each profile had the same probability of appearing, $1/823,543 \approx 1.21 \times 10^{-6}$ –close to one in a million, the observed distribution of the numbers of IPP will be very flat; i.e., we will expect that the realized number of IPP will be very close to the number of genes studied, 22,374, because the probability of finding two or more identical IPP will be vanishingly small; for example, the probability of finding k identical IPP will be close to $(1/823,543)^k$,

and with $k = 69$ we have $(1/823,543)^k = 0$ (we used that value of k because it happens to appear as the number of IPP realized). The formula presented for the probability of obtaining k identical IPP is not exact, because that probability in fact depends on the frequencies of pairs of models, but in any case the probabilities of finding groups of identical IPP is still **very** small under the hypothesis of randomness of patterns.

To study IPP we calculated, for each one of the i genes at each one of the two crosses, $\mathbf{C}_1$, $\mathbf{C}_2$, the number of distinct inheritance patterns observed, say $u_i \in \{1, 2, \cdots, 7\}$. Note that if $u_i = 1$ the gene will be fully consistent, having exactly the same pattern in all 7 development times, while in the other extreme, when $u_i = 7$ the gene will be as "inconsistent" as possible, having a different pattern at each expression time. We then designed an *ad hoc* function which gives values of consistency, say

$$c_i(u_i): \quad c_i(1) = 1.00, c_i(2) = 0.83, c_i(3) = 0.67, c_i(4) = 0.50, c_i(5) = 0.33, c_i(6) = 0.17, c_i(7) = 0.00$$

i.e., the consistency, measured by $c_i(u_i)$ is inversely related with u_i and it gives equally spaced intervals between the extremes $c_i(1) = 1$ –fully consistent gene, and $c_i(7) = 0$ –fully inconsistent genes. It is important to note that under the hypothesis of randomness of inheritance patterns the probability $P[u_i = 7] \approx 1$; i.e., the probability of having seven different patterns of inheritance in the seven sampled times will be close to one, and consequently the probability of observing "fully inconsistent gene patterns", $P[c_i(u_i) = 0] = P[u_i = 7] \approx 1$, that is, if the inheritance patterns are random then we will expect that the majority of the genes will present a fully inconsistent pattern.

Figure 5 presents a bar plot with the distribution of the percentages for the values of consistency, $c_i(u_i)$, while Figure 6 presents a heat map of the bi-variate distribution in percentages of $c_i(u_i)$ in crosses $\mathbf{C}_1$ (rows) and $\mathbf{C}_2$ (columns).

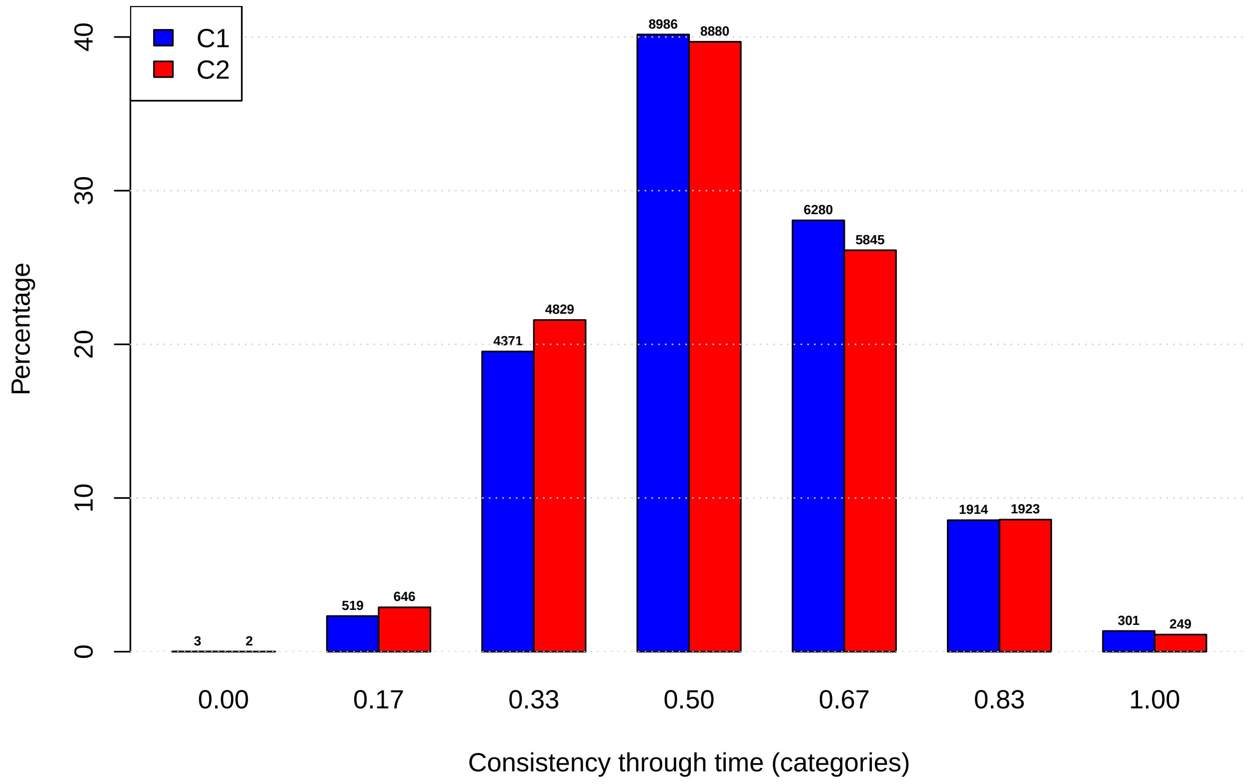

FIGURE 5. Distribution of coefficients of consistency, $c_i(u_i)$, for the 22374 at each cross, $\mathbf{C}_1$ (blue bars) and $\mathbf{C}_2$ (red bars). The X-axis present the possible values of $c_i(u_i)$, while the scale in the Y-axis is the percentage of each category. Numbers above the bars are the raw numbers of cases found.

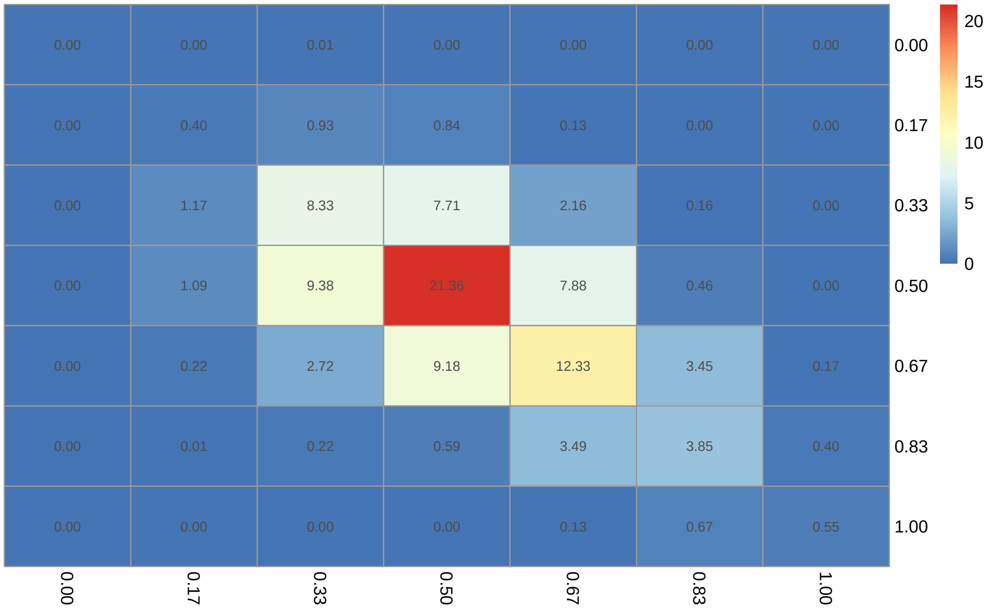

FIGURE 6. Bivariate distribution of coefficients of consistency, $c_i(u_i)$, as a heat map of percentages. X-axis are $c_i(u_i)$ in $\mathbf{C}_1$ while Y-axis are $c_i(u_i)$ in $\mathbf{C}_2$. Cell values are percentages of genes with reference to the total of 22374 that present the values each combination of values in both crosses. Pearson's product-moment correlation, $\hat{r} = 0.5766$ between values in the two crosses.

Figure 5 shows that the number of cases for which we have $c_i(u_i) = 0$ are extremely small, 3 and 2 for crosses $\mathbf{C}_1$ and $\mathbf{C}_2$, respectively. These correspond to percentages of approximately 0.01% in both cases. Thus we can fully discard the hypothesis that inheritance patterns are fully random through time. In contrast, we see that for the majority of the genes there is a strong tendency to have similar proportion of inheritance patterns, i.e., values of $c_i(u_i) > 0$, given that we have (for both crosses) estimated frequencies $\hat{P}[c_i(u_i) > 0] \approx 0.9999$, that is the great majority of genes have consistencies larger than 0. In fact, in Figure 5 we see that $\approx 40\%$ of the consistencies through time are $c_i(u_i) = 0.5$, and the distributions in both crosses are very alike, having larger percent values for cases at the right hand side of the distribution, $c_i(u_i) = 0.67$, $c_i(u_i) = 0.63$ and $c_i(u_i) = 1$, than for the cases at the left hand side of the distribution; $c_i(u_i) = 0.33$, $c_i(u_i) = 0.17$ and $c_i(u_i) = 0$, respectively. This demonstrates that there is a tendency to have the same inheritance pattern through time in both crosses.

Figure 6 presents the bivariate distribution of the percentages of cases $c_i(u_i)$ in cross $\mathbf{C}_1$ (X-axis) and $\mathbf{C}_2$ (Y-axis). We can see that in $\approx 21.36\%$ we have values of consistency of 0.5 for both crosses. That percentage is in excess to the one expected under fully independent univariate distributions, which is 15.94%. Correlation between the consistencies in both crosses is $\hat{r} = 0.5766$, which implies that approximately 33% of the consistency variance in one cross can be explained by the variance in the other cross ($\hat{r}^2 = 0.3325$).

We know that the possible numbers of IPP ("Inheritance Pattern Profile") is $7^7 = 823,543$, however the number of different IPP observed are 14,262 and 16,259 for crosses $\mathbf{C}_1$ and $\mathbf{C}_2$, respectively; i.e., less than 2% of the possible IPP are observed in each cross. This is in agreement with the fact that the IPP are not "random" –as shown above by the distributions of the consistency values. Of particular interest are fully consistent IPP, i.e., genes that have a single inheritance pattern all along the development of the fruit. In Figure 5 we have seen that there are 301 and 249 of such fully consistent genes for crosses $\mathbf{C}_1$ and $\mathbf{C}_2$, respectively (bars at the right hand side in that figure). Figure 7 shows a Venn diagram with the numbers in the sets of fully consistent genes in crosses $\mathbf{C}_1$ and $\mathbf{C}_2$.

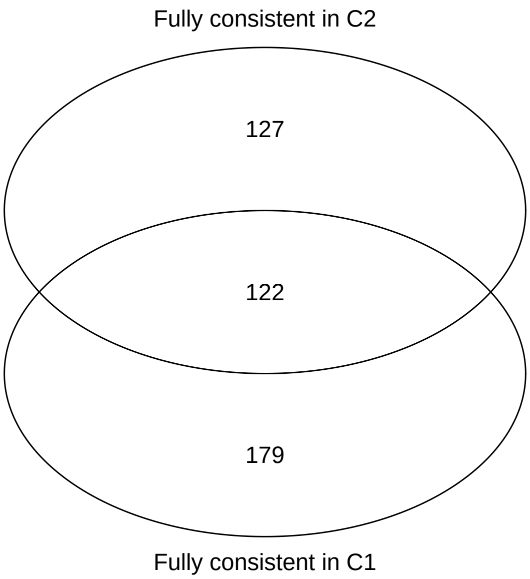

FIGURE 7. Venn diagram showing the numbers of elements in the subsets formed by sets "Fully consistent" genes in each cross. Note: |Fully consistent in $\mathbf{C}_1$| = 301; |Fully consistent in $\mathbf{C}_2$| = 249; |Fully consistent in $\mathbf{C}_1 \cup$ Fully consistent in $\mathbf{C}_2$| = 428 and |Fully consistent in $\mathbf{C}_1 \cap$ Fully consistent in $\mathbf{C}_2$| = 122.

To be fully consistent a gene must have a single one of the possible patterns in all and every one of the 7 states of development (0, 10, $\cdots$, 60 DAA). Thus, there are only 7 different possibilities for fully consistent genes, i.e., the one presenting $P_1 = F_1 = P_2$ ($m_n = 0$), $\cdots$, $P_2 < F_1 < P_1$ ($m_n = 6$). Let's use the following notation for the sets of consistent genes at each one of the two crosses, say, $\mathcal{C}_1$: {Fully consistent genes in $\mathbf{C}_1$}. $\mathcal{C}_2$: {Fully consistent genes in $\mathbf{C}_2$}. Table 8 presents the number of fully consistent genes classified by inheritance pattern at each relevant set.

TABLE 8. Number of fully consistent genes by set and inheritance pattern.

Set	$P_1 < F_1 < P_2$	$P_1 < P_2 < F_1$	$F_1 < P_1 < P_2$	$F_1 < P_2 < P_1$	$P_2 < P_1 < F_1$	$P_2 < F_1 < P_1$	$\sum$
$\mathcal{C}_1$ in $\mathbf{C}_1$	182	0	3	1	1	114	301
$\mathcal{C}_2$ in $\mathbf{C}_2$	123	7	3	3	8	105	249
$\mathcal{C}_1 \cap \mathcal{C}_2$ in $\mathbf{C}_1$	70	0	0	0	0	52	122
$\mathcal{C}_1 \cap \mathcal{C}_2$ in $\mathbf{C}_2$	69	0	1	0	0	52	122

It is important to notice that the inheritance pattern $P_1 = F_1 = P_2$ is not present in Table 8, and this is because there were no cases in which such pattern was consistently present in all development times, i.e., even when particular genes could follow such pattern –not being expressed in a given time, the pattern was not repeated during all times. On the other hand, the large majority of the patterns in fully consistent genes are of type "Intermediate F_1", say $P_1 < F_1 < P_2$ and $P_2 < F_1 < P_1$, where the value of the F_1 is between the ones of the parents. These inheritance patterns represent between 92 and 100% of the sums of fully consistent patterns in each one of the 4 sets considered in Table 8. GO enrichment analyses using the 122 genes fully consistent in both crosses (set $\mathcal{C}_1 \cap \mathcal{C}_2$) as target set did not give any BP, MF or CC enriched at FDR of 5%.

We finish this section by presenting plots of the standardized expression of genes which are in the two extremes of the distribution of $c_i(u_i)$ (Figure 5). Figure 8 presents a case where $c_i(u_i) = 0$, while Figures 9 and 10 present a case where $c_i(u_i) = 1$ in both crosses, $\mathbf{C}_1$ and $\mathbf{C}_2$. Those Figures were obtained with the function "`cross.plot()`" of the *ChiliCross* package. For interpretation of these figures it is important to remember that the the plots present Standardized Gene Expression, i.e., the observed **model** of inheritance, while the annotation given below each one of the seven rows corresponds to the **sub-model**, i.e., the inference including statistical tests (See section S4).

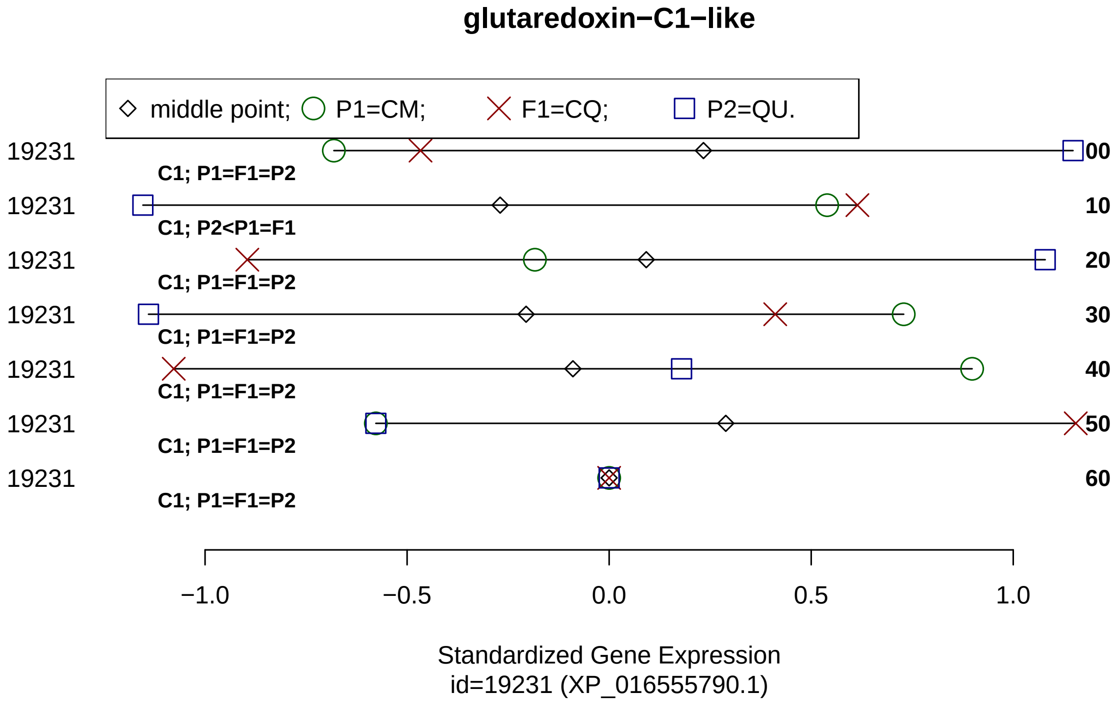

FIGURE 8. Example of expression patterns for a gene which has fully inconsistent gene patterns ($c_i(u_i) = 0$) in cross $\mathbf{C}_1$. R command: "`cross.plot(x = C1[C1$id == 19231, ], cross = "C1")`"

Figure 8 present one of the extremely unusual cases where $c_i(u_i) = 0$ –there are only 3 cases of $c_i(u_i) = 0$ in cross $\mathbf{C}_1$, i.e., the relative observed frequency of genes with this full inconsistency is $3/22374 \approx 0.0001$ in $\mathbf{C}_1$. In this figure we can observe that the expression patterns (plot gene expression) at each one of the times of development are different; in fact, each one of the times of development show one of the seven different patterns, producing a value of the coefficient of time consistency, $c_i(u_i)$, equal to 0, even when some of the sub-models –annotations below each row, are equal. If inheritance patterns were random through time, the value $c_i(u_i) = 0$ will be the most common, and in opposition to this hypothesis, the values of $c_i(u_i) = 0$ are the rarest (see Figure 5).

Figures 9 and 10 exemplify a case where $c_i(u_i) = 1$ in both crosses, $\mathbf{C}_1$ and $\mathbf{C}_2$. This is one of the 122 cases (relative frequency $122/22374 \approx 0.005$) of fully consistent genes in both crosses, i.e., genes that have exactly the same inheritance model (in this case $P_1 < F_1 < P_2$; see the plot of standardized gene expression) in all 7 times of development, even when the sub-models at time 0 DAA are different, because the difference between F_1 and P_2 did not reach significance. Under the hypothesis of fully random inheritance patterns, these cases will have a **very** small probability, approximately equal to $(1/7^7)^7 \approx 3.9 \times 10^{-42}$. Thus, the happening of these 122 cases in a frequency ≈ 0.005, which is 1.4×10^{39} times larger that the one expected under random assignation of expression patterns allows the sure rejection of the random allocation hypothesis.

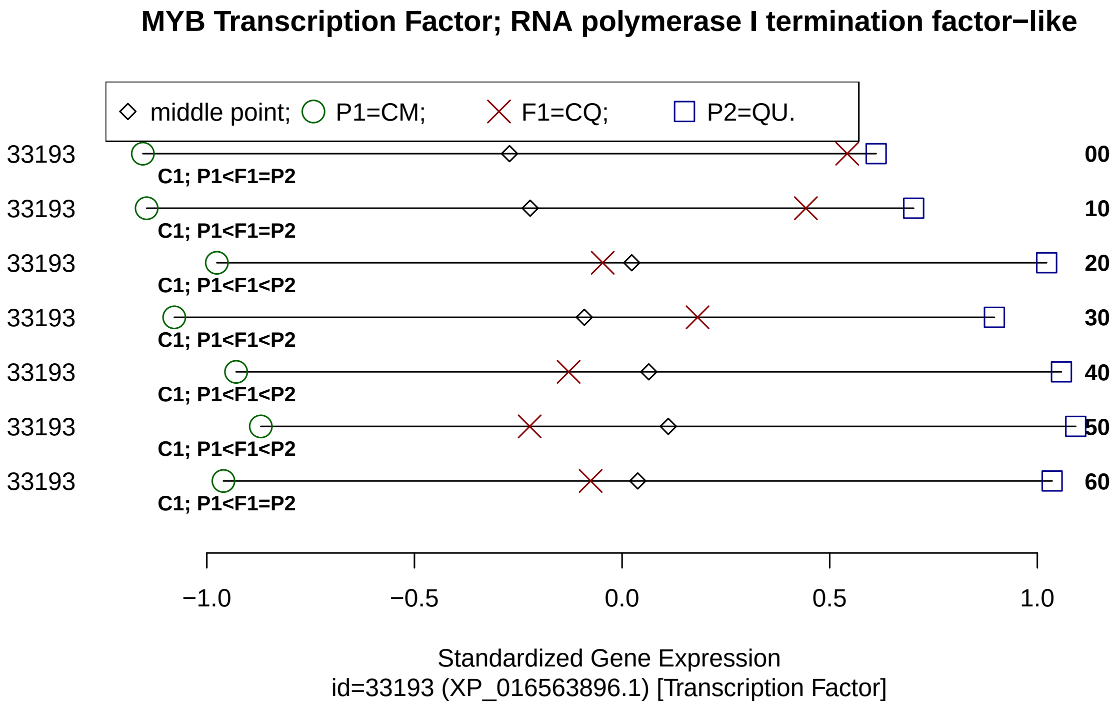

FIGURE 9. Example of expression patterns for a gene which has fully consistent gene patterns ($c_i(u_i) = 1$) in cross $\mathbf{C}_1$. R command: "`cross.plot(x = C1[C1$id == 33193, ], cross = "C1")`"

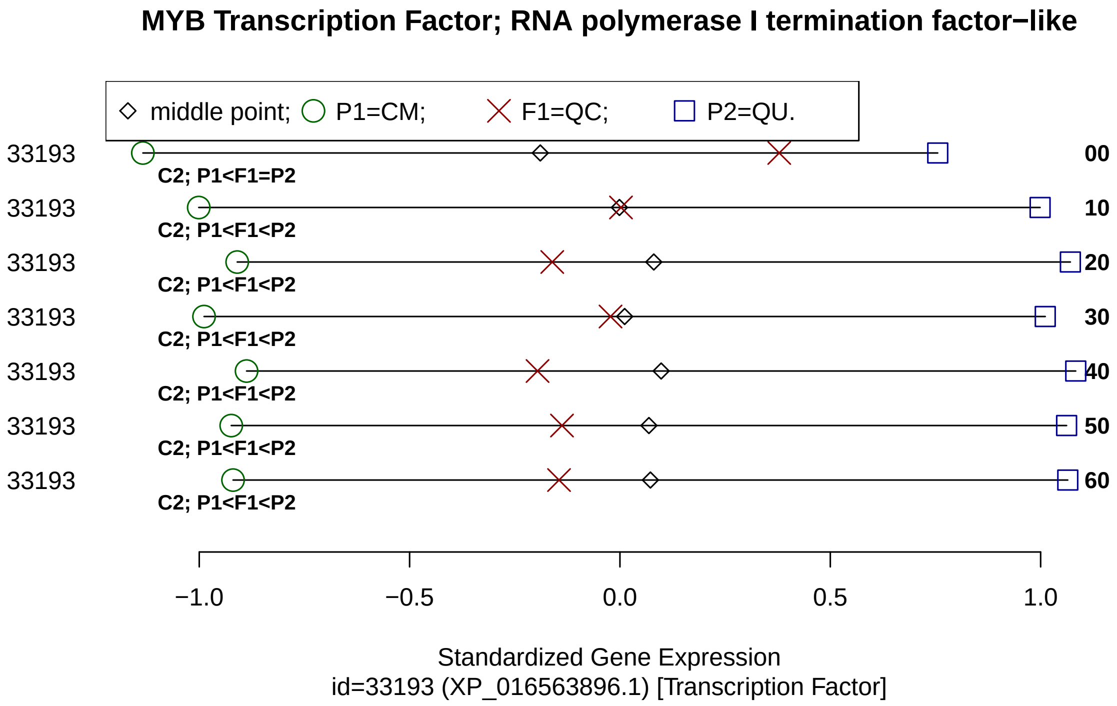

FIGURE 10. Example of expression patterns for a gene which has fully consistent gene patterns ($c_i(u_i) = 1$) in cross $\mathbf{C}_2$. R command: "`cross.plot(x = C2[C2$id == 33193, ], cross = "C2")`"

From this section we can conclude that there is a strong tendency of the genes to show a persistent expression pattern throughout fruit development, and such tendency is far away from the one expected by pure chance.

S3. Gene Ontology (GO) analyses

The **Gene Ontology Resource** (GO) provides a controlled vocabulary in the aspects of biological process (BP), molecular function (MF) and cellular component (CC) (1), and **Gene Ontology Enrichment** analysis (9) performs a Fisher's exact test on a 2×2 contingency table to evaluate if a set of target genes is "enrich" (augmented) or "depleted" (decreased, "impoverished") of genes which are categorized in a particular GO aspect. Such test gives the odds for the table, and odds values > 1 indicate "enrichment" while odds values < 1 indicate decreased number of annotated genes in the target set. Also a p-value for the test is obtained to judge the significance of the results. Our R package "*Salsa*" (4; 7) allows this analyses for any arbitrary set of genes.

Here we describe the GO enrichment analyses performed within the *Salsa* package. Those results are available for data-mining as part of the *ChiliCross* (5) package (see Section S4).

We have a total of 19 sub-models, estimated at 7 times of fruit development and in two crosses, $\mathbf{C}_1$ and $\mathbf{C}_2$. This classification schema defines a total of $19 \times 7 \times 2 = 266$ gene sets of variable sizes. The next box of R commands and results shows statistics for the numbers of genes in each relevant set.

```
--------------------------------------------------------------------------------------
> library(Salsa)
> library(ChiliCross)
> length(unique(C1$id)) # Number of different genes expressed in cross 1.
[1] 22374
> length(unique(C2$id)) # Number of different genes expressed in cross 2.
[1] 22374

# Table for the number of genes in each one of the 19*7=133 sets of
# different genes in each time x sub-model combination in cross C1
 > table(C1$sub.model, C1$time)

             00    10    20    30    40    50    60
  F1<P1<P2   279     7     7    35    10   368     6
  F1<P1=P2   492    11    18   141   360  1077    17
  F1<P2<P1   209    13    19   139    16   203     2
  F1<P2=P1   359    29    58   202  1010   603    11
  F1=P1<P2   205   759   273   292   284  1611   198
  F1=P2<P1   169   137   311   751    66   862   176
  P1<F1<P2   191   324   341   551    94   877    11
  P1<F1=P2   647   177   482  1605    80  1151   150
  P1<P2<F1    44    13     9   168    17   159     0
  P1<P2=F1   738   153   295  1215    26   492   323
  P1=F1<P2   390  1586   841   562  1039  1550    58
  P1=F1=P2 14250 16003 18201 14549 18478 11760 21195
  P1=P2<F1   257    52    80   245   146   154     7
  P2<F1<P1   116   137   195   321    68   210     8
  P2<F1=P1   899  2167   619   427   383   177    96
  P2<P1<F1   156     4    12    29     6    59     1
  P2<P1=F1  2346   706   260   191    90   123    71
  P2=F1<P1   127    91   337   823   125   765    39
  P2=P1<F1   500     5    16   128    76   173     5

# Statistics for the number of different genes at each combination.
> summary(as.vector(table(C1$sub.model, C1$time)))
```

```
   Min. 1st Qu.  Median    Mean 3rd Qu.    Max.
      0      58     177    1178     500   21195
> temp <- as.vector(table(C1$sub.model, C1$time))
> length(temp)
[1] 133
> length(temp[temp>=5])
[1] 129

# Table for the number of genes in each one of the 19*7=133 sets of
# different genes in each time x sub-model combination in cross C2
> table(C2$sub.model, C2$time)

              00    10    20    30    40    50    60
  F1<P1<P2   505   748  1165   391   133   829   140
  F1<P1=P2   709   277  2518   899   702   424   311
  F1<P2<P1    55    96   396   362     2    47    10
  F1<P2=P1   279    87  1584   477   103   147    69
  F1=P1<P2     0   119     2     2     3   106     2
  F1=P2<P1   156   717   485   752    58   431   106
  P1<F1<P2   484   297   363   700    37   916   122
  P1<F1=P2  1164   412   438  1561   161  2077   645
  P1<P2<F1   116    93   300   315     3    86    13
  P1<P2=F1   655   550   326  1178    88   566   231
  P1=F1<P2   447   775  1322   729   231  1017   315
  P1=F1=P2 10449 15359  9865 12221 20230 13319 19031
  P1=P2<F1   879   222   849   493    40   121    61
  P2<F1<P1   489   198   381   414    19   241    67
  P2<F1=P1  1229  1085   710   420   231   257   347
  P2<P1<F1   689    24   159    70     0    45     5
  P2<P1=F1  1450   692   375   173    63   246   125
  P2=F1<P1   400   338   493   780    61   931   183
  P2=P1<F1  2137    84   504   215     7   204    51

# Statistics for the number of different genes at each combination.
> summary(as.vector(table(C2$sub.model, C2$time)))
   Min. 1st Qu.  Median    Mean 3rd Qu.    Max.
      0     103     326    1164     709   20230
> temp <- as.vector(table(C2$sub.model, C2$time))
> length(temp[temp>=5])
[1] 125
--------------------------------------------------------------------------------
```

As seen from the previous box, the number of different genes per combination of sub-model $\times$ time of development presents a high variation, being between 0 and 21,195 with a median of 177 for cross $\mathbf{C}_1$ (data frame `C1`), and between 0 and 20,230 with a median of 326 for cross $\mathbf{C}_2$ (data frame `C2`). Given that the statistical power of the Fisher's exact test used for the GO enrichment analysis depends on the number of genes in the target set, we decided to perform such analyses only for the cases where the target set was ≥ 5. This included 129 gene sets for $\mathbf{C}_1$ and 125 gene sets for $\mathbf{C}_2$ (see box above).

GO enrichment analyses was performed for all annotated Biological Processes (BP), Molecular Functions (MF) and Cell Components (CC) in all gene sets ≥ 5 for each one of the two crosses. The analyses were performed using the *Salsa* function "`analyze.all.GO()`", filtering results by a FDR $\leq$ 10%. A second filter imposed to the results was to have an estimated odd value $>$ 1 –thus, at the end we have

only enriched cases with a FDR ≤ 10%. Those results are available for data-mining in the *ChiliCross* package, data frames `GO.enr.BP`, `GO.enr.MF` and `GO.enr.CC` for BP, MF and CC, respectively. The next box presents summaries of statistics for those data frames.

```
--------------------------------------------------------------------------------------
# Summaries for Biological Processes (BP)
> ? GO.enr.BP # Review the help for the data frame (not shown)
> nrow(GO.enr.BP) # Number of results in the data.frame
[1] 3096
> summary(GO.enr.BP$odds)
   Min. 1st Qu.  Median    Mean 3rd Qu.    Max.
  1.095   1.356   1.661     Inf   2.768     Inf
> summary(GO.enr.BP$Q)
     Min.   1st Qu.    Median      Mean   3rd Qu.      Max.
0.000e+00 4.600e-07 4.089e-03 2.245e-02 3.917e-02 9.983e-02
> length(unique(GO.enr.BP$aspect.id)) # Different BPs
[1] 580
# BPs annotated by combination of time and class
> table(GO.enr.BP$time, GO.enr.BP$s.class)

     High F1 Intermediate F1 Low F1 P1=F1=P2
  00      21              19     94      283
  10       6              19     10      439
  20      11               9    154      341
  30       0               8      8      357
  40       2               0     16      400
  50      10              69     50      309
  60      12               6      0      443

 > # Summaries for Molecular Function (MF)
> ? GO.enr.MF # Review the help for the data frame (not shown)
> nrow(GO.enr.MF) # Number of results in the data.frame
[1] 1772
> summary(GO.enr.MF$odds) # Odds statistics
   Min. 1st Qu.  Median    Mean 3rd Qu.    Max.
  1.100   1.531   2.276     Inf   4.322     Inf
> summary(GO.enr.MF$Q) # Q-value statistics
    Min.  1st Qu.   Median     Mean  3rd Qu.     Max.
0.000000 0.001207 0.015270 0.028008 0.051086 0.099733
> length(unique(GO.enr.MF$aspect.id)) # Different MFs
[1] 368
> # MFs annotated by combination of time and class
> table(GO.enr.MF$time, GO.enr.MF$s.class)

     High F1 Intermediate F1 Low F1 P1=F1=P2
  00      10              11     57      116
  10       4              91     12      227
  20      15              54     40      139
  30       7              41     11      131
  40      10               4     26      268
  50      22              34     25      127
  60       7               8      0      275
```

```
 > # Summaries for Cell Component (CC)
> ? GO.enr.CC # Review the help for the data frame (not shown)
> nrow(GO.enr.CC) # Number of results in the data.frame
[1] 374
> summary(GO.enr.CC$odds) # Odds statistics
   Min. 1st Qu.  Median    Mean 3rd Qu.    Max.
  1.122   1.318   1.679     Inf   3.507     Inf
> summary(GO.enr.CC$Q) # Q-value statistics
     Min.   1st Qu.    Median      Mean   3rd Qu.      Max.
0.0000000 0.0007907 0.0149848 0.0295544 0.0575503 0.0999101
> length(unique(GO.enr.CC$aspect.id)) # Different CCs
[1] 91
> # CCs annotated by combination of time and class
> table(GO.enr.CC$time, GO.enr.CC$s.class)

     High F1 Intermediate F1 Low F1 P1=F1=P2
  00       7               0     17       24
  10       4              18      6       33
  20       4               2     18       29
  30       4               7      1       60
  40       1               0      0       45
  50       2               9      0       46
  60       0               0      3       34

# Total of results
> nrow(GO.enr.BP)+nrow(GO.enr.MF)+nrow(GO.enr.CC)
[1] 5242
```

From the results in the above box we can appreciate the richness and heterogeneity of the GO enrichment results. The number of different aspects enriched is of 580 BPs, 368 MFs and 91 CCs, with a total of 5,242 GO enrichment results which are heterogeneously distributed among aspects (BP, MF or CC), in two crosses ($\mathbf{C}_1$ and $\mathbf{C}_2$) and in combinations of inheritance sub-model $\times$ time of fruit development.

A complication to interpret GO enrichment analysis is that genes are usually annotated in more than one category within the ontology tree of relations, and this implies that GO enriched terms are not statistically independent. To mitigate this dependence, within the sets of enriched BP terms at each time $\times$ inheritance combination, we selected pairs of terms that were not sharing annotated genes, reporting only the independent terms with the largest number of annotated genes in the corresponding category. Table 9 presents the result of this exercise of selection of enriched BP terms, demonstrating the capabilities of data mining within the *ChiliCross* package.

Table 9. Enriched and independent BP per time of development and pattern of inheritance.

Time	Pattern	Description	GO	Odds
00	$P_1 < P_2 < F_1$	translation	**GO:0006412**	2
00	$F_1 < P_2 < P_1$	plasma membrane fusion	**GO:0045026**	17
00	$F_1 < P_2 < P_1$	exocytosis	**GO:0006887**	6
00	$F_1 < P_2 < P_1$	GPI anchor metabolic process	**GO:0006505**	4
00	$F_1 < P_1 < P_2$	cell wall modification	**GO:0042545**	4
00	$F_1 < P_1 < P_2$	external encapsulating structure organization	**GO:0045229**	3
00	$F_1 < P_1 < P_2$	cell wall organization	**GO:0071555**	3
00	$P_2 < P_1 < F_1$	nitrogen fixation	**GO:0009399**	21
00	$P_2 < P_1 < F_1$	regulation of translation	**GO:0006417**	2
00	$P_2 < P_1 < F_1$	posttranscriptional regulation of gene expression	**GO:0010608**	2
10	$P_1 < P_2 < F_1$	transition metal ion homeostasis	**GO:0055076**	19
10	$F_1 < P_2 < P_1$	regulation of translation	**GO:0006417**	7
10	$P_2 < F_1 < P_1$	response to radiation	**GO:0009314**	6
10	$P_2 < F_1 < P_1$	DNA-templated transcription, initiation	**GO:0006352**	3
10	$P_2 < F_1 < P_1$	negative regulation of cellular process	**GO:0048523**	2
20	$F_1 < P_2 < P_1$	DNA alkylation	**GO:0006305**	9
30	$P_1 < F_1 < P_2$	mitotic sister chromatid segregation	**GO:0000070**	6
30	$P_1 < F_1 < P_2$	sister chromatid segregation	**GO:0000819**	6
30	$P_1 < F_1 < P_2$	mitotic nuclear division	**GO:0007067**	3
30	$P_1 < P_2 < F_1$	ceramide metabolic process	**GO:0006672**	13
30	$P_1 < P_2 < F_1$	single-organism carbohydrate catabolic process	**GO:0044724**	3
30	$P_1 < P_2 < F_1$	ribonucleoside diphosphate metabolic process	**GO:0009185**	3
30	$P_1 < P_2 < F_1$	ADP metabolic process	**GO:0046031**	3
40	$P_1 < F_1 < P_2$	polyol metabolic process	**GO:0019751**	8
40	$P_1 < F_1 < P_2$	DNA-templated transcription, initiation	**GO:0006352**	3
40	$F_1 < P_1 < P_2$	regulation of translation	**GO:0006417**	3
40	$F_1 < P_1 < P_2$	posttranscriptional regulation of gene expression	**GO:0010608**	3
40	$F_1 < P_1 < P_2$	regulation of cellular amide metabolic process	**GO:0034248**	3
40	$F_1 < P_1 < P_2$	microtubule-based movement	**GO:0007018**	3
50	$P_1 < F_1 < P_2$	nucleoside diphosphate metabolic process	**GO:0009132**	3
50	$P_1 < F_1 < P_2$	RNA methylation	**GO:0001510**	3
50	$P_1 < P_2 < F_1$	response to oxidative stress	**GO:0006979**	4
50	$F_1 < P_1 < P_2$	regulation of translation	**GO:0006417**	7
60	$P_1 < P_2 < F_1$	RNA polyadenylation	**GO:0043631**	7
60	$P_1 < P_2 < F_1$	methylation	**GO:0032259**	3
60	$P_1 < P_2 < F_1$	regulation of cellular amide metabolic process	**GO:0034248**	3
60	$P_1 < P_2 < F_1$	regulation of protein metabolic process	**GO:0051246**	2
60	$P_2 < F_1 < P_1$	pyruvate metabolic process	**GO:0006090**	4
60	$P_2 < F_1 < P_1$	nucleoside diphosphate phosphorylation	**GO:0006165**	4
60	$P_2 < F_1 < P_1$	nucleoside diphosphate metabolic process	**GO:0009132**	4
60	$P_2 < F_1 < P_1$	pyridine-containing compound metabolic process	**GO:0072524**	3

S4. The "*ChiliCross*" R package

The R package "*ChiliCross*" (5) was programed to facilitate data-mining of the results presented in the paper. You can download the package from **ChiliCross**. After installing the package you could include it in your R environment with command "`library(ChiliCross)`". To follow the results presented here you need to install and load the package.

The package contains standardized gene expression for two crosses of chili pepper (*Capsicum annuum* L.) during fruit development and results for Gene Ontology enrichment analyses for sets of genes. There is also a function to plot cross results. Table 10 presents the data frames contained in *ChiliCross* with a brief description.

Table 10. Data frames in package "*ChiliCross*".

Name	Brief description
`all.models`	A simple data frame with auxiliar variables to describe characteristics of classes, models and sub-models.
`C1`	Results for cross $\mathbf{C}_1$ (C1), including standardized expressions for each cross participant. Each row has results for a gene (`id`) at a particular time of fruit development (`time`).
`C2`	Results for cross $\mathbf{C}_2$ (C2), including standardized expressions for each cross participant. Each row has results for a gene (`id`) at a particular time of fruit development (`time`).
`gene`	For each one of the expressed genes (rows) this data frame contains protein identifiers and descriptions.
`GO.enr.BP`	Gene Ontology (GO) enrichment results for Biological Processes (BPs) for 133 sets of genes. Maximum FDR 10%.
`GO.enr.MF`	Gene Ontology (GO) enrichment results for Molecular Function (MFs) for 133 sets of genes. Maximum FDR 10%.
`GO.enr.CC`	Gene Ontology (GO) enrichment results for Cell Component (CCs) for 133 sets of genes. Maximum FDR 10%.
`results.key`	A remainder of participants of the crosses useful for users.

The main results of data curation presented in Table 10 can be divided into categories: Main results (data frames `C1` and `C2`) containing standardized expressions for each combination of gene × time of fruit development in crosses $\mathbf{C}_1$ and $\mathbf{C}_2$, respectively; Gene Ontology (GO) enrichment results (data frames `GO.enr.BP`, `GO.enr.MF` and `GO.enr.CC`) and auxiliar data frames (`all.models`, `gene` and `results.key`). Genes are identified by the unique numeric variable "`id`", which is present in data frames `C1`, `C2` and `gene`.

S4.1. **Brief tour into the package.** In the following box we present a commented tour that will familiarize you with the package. You can reproduce this box by entering the orders after the R prompt ("`>`") –you can just copy and paste the corresponding lines. Comments (any text following "`#`") explain the results. Not all results are shown.

```
------------------------------------------------------------------------------------
> library(Salsa) # See the Note at the beginning about this package.
> library(ChiliCross) # Load the package.

> ? ChiliCross # Ask help for the package (not shown).
# (You could follow the examples in that help window)

# First see the definition of the crosses
> results.key # A very simple data.frame
                        cross P1 P2 F1
```

```
1 C1: CM female x QU male = CQ CM QU CQ
2 C2: QU female x CM male = QC CM QU QC

# The schema of inheritance classification
> ? all.models # Help about that data.frame
> nrow(all.models) # Number of rows of the data.frame
[1] 19
> head(all.models) # First 6 rows of the data.frame
  sub.model mi    model  class is.main
1  P1=F1=P2  0 P1=F1=P2   NULL   FALSE
2  F1<P1<P2  1 F1<P1<P2 Low F1    TRUE
3  F1<P1=P2  1 F1<P1<P2 Low F1   FALSE
4  F1=P1<P2  1 F1<P1<P2 Low F1   FALSE
5  F1<P2<P1  2 F1<P2<P1 Low F1    TRUE
6  F1<P2=P1  2 F1<P2<P1 Low F1   FALSE

> table(all.models$class) # See also Fig. 1 in main text

        High F1 Intermediate F1          Low F1            NULL
              6               6               6               1

> ? gene # See the help for that auxiliar data.frame
# (that data.frame exist in both, Salsa and ChiliCross)

> table(gene$isTF) # How many annotated as TF (Transcription Factor)?

FALSE  TRUE
28127  1792

> head(gene[gene$isTF==T,]) # Some of the TFs
     id         ProtId
32   32 XP_016557439.1
52   52 XP_016539951.1
53   53 XP_016545756.1
75   75 XP_016572804.1
104 104 XP_016582323.1
156 156 XP_016557195.1
                                                             Prot.Desc isTF
32                                          protein RADIALIS-like 1 TRUE
52                                     myb-related protein 308-like TRUE
53  single-stranded DNA-bindig protein WHY2, mitochondrial isoform X1 TRUE
75                  putative zinc finger protein At1g68190 isoform X2 TRUE
104                              uncharacterized protein LOC107879906 TRUE
156                  probable WRKY transcription factor 69 isoform X1 TRUE

> ? C1 # See the help for that data.frame (not shown here)
> head(C1, 2)
  id time mi    model   class C1.left C1.right sig.left sig.right
1  3   00  2 F1<P2<P1  Low F1   QU.CQ    CM.QU    FALSE     FALSE
2 19   00  6 P2<P1<F1 High F1   CM.QU    CM.CQ    FALSE     FALSE
    SE.P1.CM  SE.F1.CQ   SE.P2.QU sub.model
1  0.6051573 -1.154247  0.5490894  P1=F1=P2
```

```
2 -0.1726369  1.075079 -0.9024421  P1=F1=P2
# And the same for C2
> head(C2, 2)
  id time mi    model   class C2.left C2.right sig.left sig.right
1  3   00  2 F1<P2<P1  Low F1   QU.QC    CM.QU     TRUE     FALSE
2 19   00  6 P2<P1<F1 High F1   CM.QU    CM.QC    FALSE      TRUE
    SE.P1.CM  SE.F1.QC   SE.P2.QU sub.model
1  0.5978674 -1.154455  0.5565872  F1<P2=P1
2 -0.0817478  1.038365 -0.9566169  P2=P1<F1

# Assume that you are interested in WRKY TFs,
# How many of those are annotated in data.frame 'gene'?
# First, get the id of genes that contain 'WRKY' in description
> ids.WRKY <- gene$id[grep('WRKY',gene$Prot.Desc)]
> length(ids.WRKY) # How many of those?
[1] 71
# Check that all of them are annotated as TFs:
> nrow(gene[is.element(gene$id, ids.WRKY)&(gene$isTF==T),])
[1] 71
# See some of the descriptions of proteins coded by those genes
> head(gene$Prot.Desc[is.element(gene$id, ids.WRKY)])
[1] "probable WRKY transcription factor 69 isoform X1"
[2] "probable WRKY transcription factor 31 isoform X2"
[3] "probable WRKY transcription factor 70"
[4] "probable WRKY transcription factor 7"
[5] "probable WRKY transcription factor 13"
[6] "LOW QUALITY PROTEIN: WRKY transcription factor 44-like"

# Now let's tabulate the sub-models of those 71 genes in C1
> table(C1$sub.model[is.element(C1$id, ids.WRKY)])

F1<P1=P2 F1<P2<P1 F1<P2=P1 F1=P1<P2 F1=P2<P1 P1<F1<P2 P1<F1=P2 P1<P2=F1
       6        2        6        7        8        7       12       10
P1=F1<P2 P1=F1=P2 P1=P2<F1 P2<F1<P1 P2<F1=P1 P2<P1=F1 P2=F1<P1 P2=P1<F1
      21      303        3        3        9        8        5        3

# And obtain the ids of genes that are WRKY and have sub-model 'P2<F1<P1'
> ids.WRKY2 <- C1$id[is.element(C1$id, ids.WRKY)&(C1$sub.model=='P2<F1<P1')]
> ids.WRKY2 # Genes with those identifiers (id):
[1] 34824  9740 15244

# Note that we have that sub.model only at particular times
> C1[(is.element(C1$id, ids.WRKY2))&(C1$sub.model=='P2<F1<P1'),]
          id time mi    model           class C1.left C1.right sig.left
21688  34824   00  4 P2<F1<P1 Intermediate F1   QU.CQ    CM.CQ     TRUE
118017  9740   50  4 P2<F1<P1 Intermediate F1   QU.CQ    CM.CQ     TRUE
121110 15244   50  4 P2<F1<P1 Intermediate F1   QU.CQ    CM.CQ     TRUE
       sig.right SE.P1.CM   SE.F1.CQ   SE.P2.QU sub.model
21688       TRUE 1.153600 -0.5331439 -0.6204558  P2<F1<P1
118017      TRUE 1.126931 -0.3454768 -0.7814546  P2<F1<P1
121110      TRUE 1.136063 -0.3890860 -0.7469766  P2<F1<P1
```

```
# Let's see the rows of gene for those identifiers
> gene[is.element(gene$id, ids.WRKY2),]
         id        ProtId                             Prot.Desc isTF
9740   9740 XP_016556725.1 probable WRKY transcription factor 75 TRUE
15244 15244 XP_016562005.1 probable WRKY transcription factor 71 TRUE
34824 34824 XP_016581006.1 probable WRKY transcription factor 71 TRUE

# Are there other genes annotated as 'WRKY transcription factor 71'?
> ids.WRKY3 <- gene$id[grep('WRKY transcription factor 71', gene$Prot.Desc)]
> length(ids.WRKY3)
[1] 4
> gene[is.element(gene$id, ids.WRKY3),]
         id        ProtId                             Prot.Desc isTF
9858   9858 XP_016581006.1 probable WRKY transcription factor 71 TRUE
15244 15244 XP_016562005.1 probable WRKY transcription factor 71 TRUE
25805 25805 XP_016581006.1 probable WRKY transcription factor 71 TRUE
34824 34824 XP_016581006.1 probable WRKY transcription factor 71 TRUE

# In which classes are inheritance patterns of those genes in C1?
> table(C1$class[is.element(C1$id, ids.WRKY3)])

        High F1 Intermediate F1          Low F1            null
              6              10              11               1
# And also, at which times of development?
> table(C1$class[is.element(C1$id, ids.WRKY3)], C1$time[is.element(C1$id, ids.WRKY3)])

                  00 10 20 30 40 50 60
  High F1          3  0  0  0  1  1  1
  Intermediate F1  1  2  2  1  1  1  2
  Low F1           0  2  2  3  2  2  0
  null             0  0  0  0  0  0  1
-------------------------------------------------------------------------------------
```

In the previous box we briefly illustrated some of the many data-mining possibilities of the "*ChiliCross*" R package. The results concerning the Gene Ontology (GO) analyses are illustrated in Section S3 of this document. Next sub-section presents the use of the `cross.plot()` function of the package.

S4.2. **The `cross.plot()` function.** The experiment performed here was a factorial including two crosses, $\mathbf{C}_1$ and $\mathbf{C}_2$, and 7 times of fruit development $(0, 10, \cdots, 60$ DAA). For each one of the chili pepper genes we estimated and standardized their expression, performing statistical test to classify inheritance results in 19 exhaustive sub-models of inheritance. The main results are in data frames `C1` and `C2` and function `cross.plot()` can plot any seven rows of the data frames `C1` or `C2`. The selection of exactly seven rows to be plot is not completely arbitrary; it gives the possibility to see the results of all 7 times of expression in a single figure. The next box shows some examples of the use of the function, which are illustrated by Figures .

```
-------------------------------------------------------------------------------------
> ? cross.plot # Opens a window with help for the function (not shown)

# We select to plot a gene that codes for a WRKY transcription factor
> gene[gene$id==15244,]
         id        ProtId                             Prot.Desc isTF
15244 15244 XP_016562005.1 probable WRKY transcription factor 71 TRUE
```

```
# (see previous box)

# Now we select the data.frame containing the data in C1
> temp.C1 <- C1[C1$id==15244,]
> temp.C1[,c(1,2,10:13)] # Data that will be plot
           id time   SE.P1.CM   SE.F1.CQ   SE.P2.QU sub.model
9240   15244   00 0.4754262  0.6735923 -1.1490185  P2<P1=F1
31614  15244   10 0.5160520 -1.1526029  0.6365509  P1=F1=P2
53988  15244   20 1.1013023 -0.2500686 -0.8512338  P1=F1=P2
76362  15244   30 1.1546132 -0.5896067 -0.5650065  F1=P2<P1
98736  15244   40 1.1537147 -0.6181706 -0.5355441  P1=F1=P2
121110 15244   50 1.1360625 -0.3890860 -0.7469766  P2<F1<P1
143484 15244   60 0.2972615  0.8176646 -1.1149261  P2<P1=F1

> cross.plot(x=temp.C1, cross='C1') # Produces the figure shown below
--------------------------------------------------------------------------------------
```

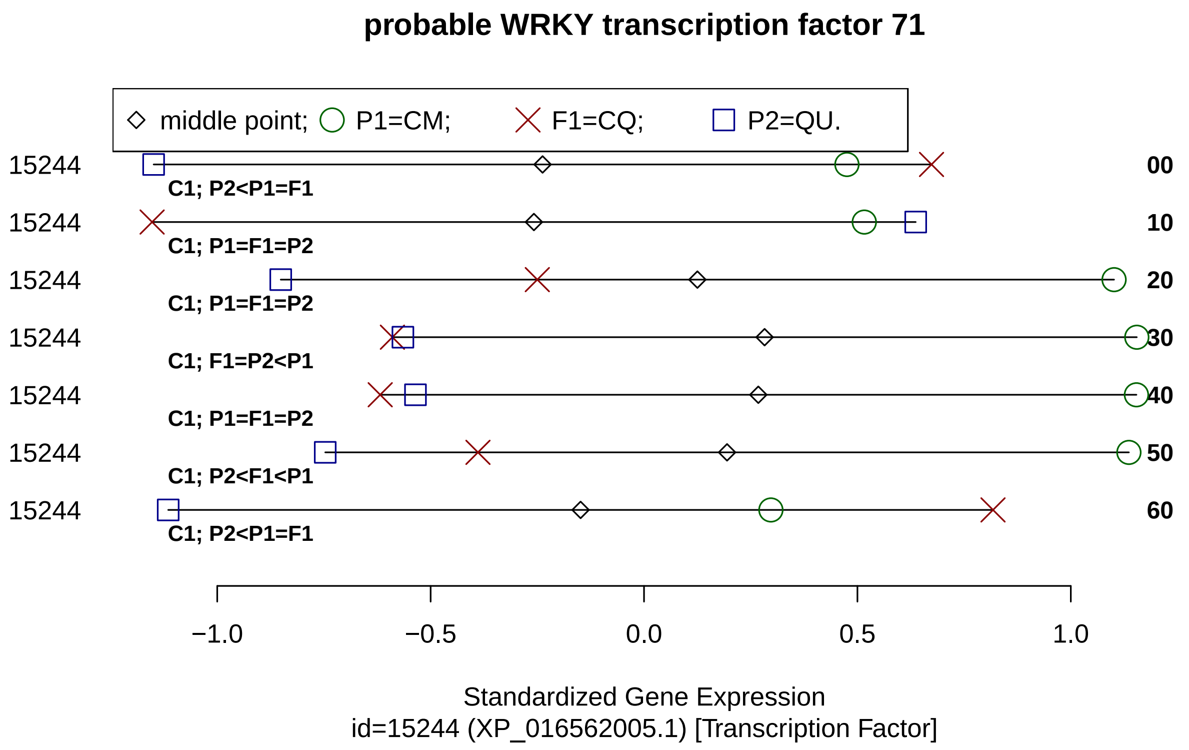

Figure 11. Figure resulting from the command "`cross.plot(x=temp.C1, cross='C1')`" (see box of R commands above).

Figures produced by "`cross.plot()`" present in the X-axis the standardized gene expression values, and in the Y-axis 7 rows, each one of them corresponding to one row of the data frame "`x`" that was passed as first input to the function as first argument. Such figures are annotated in the following way: 1 - A legend in the upper left hand right side with the definitions of the symbols employed: a diamond for the middle point between the two extremes of gene expression, a green circle indicating which accession was the P_1 parent, a red cross indicating which accession was the F_1 and a blue square indicating which accession was the P_2 parent; 2 - For each one of the 7 rows, at the left hand side margin we have the gene identifier (`id`) while at the right hand side margin we have the time of development (variable `time` in the data frame); 3 - Also, below each one of the seven rows, there is an annotation with the cross and sub-model of

the corresponding combination of gene (variable `id` in the data frame) × time of development (variable `time` in the data frame). Additionally if the data frame "`x`" that was passed as first input to the function as first argument contains **a single gene**, i.e., a unique value of variable `id`, then there will a main title in the plot, with the protein description of the gene and also a sub-title with more data for the gene.

In Figure 11 we can see the plot produced by "`cross.plot()`", when the arguments passed were

"`cross.plot(x=temp.C1, cross='C1')`".

Note that "`temp.C1`" is a data frame of seven rows containing data for the gene with `id=15244`, which is identified with `ProtId = XP_016562005.1` and `Prot.Desc` "probable WRKY transcription factor 71". Those data are automatically gathered from the `gene` data frame, given that the data frame pass to the function includes a unique gene (`id=15244`). For interpretation it is vital to take into account the annotation given below each one of the rows, that indicates the sub-model assigned given the results of the statistical tests performed. For example, in the first row of Figure 11, corresponding to `id=15244` and `time=00`, the plot of the values for the participants show the order $P_2 < P_1 < F_1$, however the annotation of that row is "**C1; P2<P1=F1**", which indicates that the data correspond to the cross $\mathbf{C}_1$ and that the **true** sub-model is $P_2 < P_1 = F_1$; i.e., while the test performed for the null hypothesis $P_2 = P_1$ was rejected, the test performed for the hypothesis $P_1 = F_1$ was not rejected. For details please see Section S1 in this document.

The box below gives further examples of the use of the "`cross.plot()`", which results are presented in Figures 12 and 13.

```
----------------------------------------------------------------------------------------
# Further examples of use of cross.plot()
# Remember that
> gene[gene$id==15244,]
         id         ProtId                             Prot.Desc isTF
15244 15244 XP_016562005.1 probable WRKY transcription factor 71 TRUE

# Obtaining the data frame in cross C2
> temp.C2 <- C2[C2$id==15244,]
# Data that will be plot
> temp.C2[,c(1,2,10:13)] # Data that will be plot
          id time   SE.P1.CM   SE.F1.QC   SE.P2.QU sub.model
9240   15244   00  0.4580340  0.6889447 -1.1469787  P2<P1=F1
31614  15244   10 -0.6733246  1.1490510 -0.4757264  P1=F1=P2
53988  15244   20  1.1340023 -0.7554929 -0.3785093  F1=P2<P1
76362  15244   30  1.1450104 -0.7017855 -0.4432249  F1<P2<P1
98736  15244   40  1.1383913 -0.4017173 -0.7366739  P1=F1=P2
121110 15244   50  1.1285057 -0.3524601 -0.7760456  P2<F1<P1
143484 15244   60  0.2335337  0.8625679 -1.0961016  P2<P1=F1

> cross.plot(x=temp.C2, cross='C2') # Produces figure (see main text)

# Are the sub-models for gene with id=15244 at each time
# (00, 10, ..., 60 DAA) equal or different in C1 and C2
> temp.C1$sub.model == temp.C2$sub.model
[1]  TRUE  TRUE FALSE FALSE  TRUE  TRUE  TRUE
> table(temp.C1$sub.model == temp.C2$sub.model)

FALSE  TRUE
    2     5
```

```
# Now lets create a data frame with the first 7 genes
# that exist in C1 at time 0 DAA
> length(C1$id[C1$time=='00']) # How many genes at time 00
[1] 22374
> temp.00 <- head(C1$id[C1$time=='00'],7) # First seven of those
> temp.00 # The identifiers
[1]  3 19 26 27 28 30 31
# What are those genes?
> gene[is.element(gene$id, temp.00), 1:3]
   id         ProtId                                              Prot.Desc
3   3 YP_009049789.1          NADH dehydrogenase subunit 6 (mitochondrion)
19 19 XP_016545450.1                      dynamin-like protein ARC5 isoform X5
26 26 XP_016545534.1 cell cycle checkpoint control protein RAD9A isoform X2
27 27 XP_016545431.1          aspartic proteinase-like protein 2 isoform X1
28 28 XP_016545540.1              phosphoglycerate mutase-like protein AT74
30 30 XP_016545761.1                      uncharacterized protein LOC107845793
31 31 XP_016545770.1                               tubby-like F-box protein 10

# Obtain a data.frame in C1 for those genes at time 0 DAA
> temp.C1.2 <- C1[(is.element(C1$id, temp.00))&(C1$time=='00'),]
> temp.C1.2[,c(1,2,10:13)] # Data that will be plot
  id time    SE.P1.CM   SE.F1.CQ   SE.P2.QU sub.model
1  3   00  0.60515732 -1.1542467  0.5490894  P1=F1=P2
2 19   00 -0.17263689  1.0750790 -0.9024421  P1=F1=P2
3 26   00 -0.61478830  1.1538730 -0.5390847  P1=F1=P2
4 27   00  0.41511883 -1.1407032  0.7255843  P1=F1=P2
5 28   00  0.23939162  0.8585775 -1.0979692  P1=F1=P2
6 30   00 -0.07382268  1.0348656 -0.9610429  P1=F1=P2
7 31   00 -1.15461259  0.5896481  0.5649645  P1=F1=P2

# And plot those data
> cross.plot(x=temp.C1.2, cross='C1') # Produces figure (see main text)
---------------------------------------------------------------------------------------
```

Figure 12 presents the result of running the function for gene with `id=15244` in the cross $\mathbf{C}_2$. It is illustrative to compare that figure with Figure 11 that presents the plot for the same gene, but in the fully independent cross $\mathbf{C}_1$.

In contrast with figures Figure 11 and Figure 13, which presented results for a single gene, Figure 13 presents the plot for different genes (identifiers in the left hand side margin of the figure) in cross $\mathbf{C}_1$ at a single time point of development: 0 DAA (annotated in the hand side margin of the figure). Thus, Figure 13 does not present a title or subtitle, because there are 7 different genes plot at a single time point. Interpretation must be done following the same guidelines than the ones given above for Figure 11.

In summary, the R package "*ChiliCross*" can be used to perform data-mining of many aspects of the standardized inheritance patterns.

RNA-Seq data are deposited in the NCBI's Gene Expression Omnibus (3), and are accessible through the GEO Series accession number GSE165448; link:

https://www.ncbi.nlm.nih.gov/geo/query/acc.cgi?acc=GSE165448

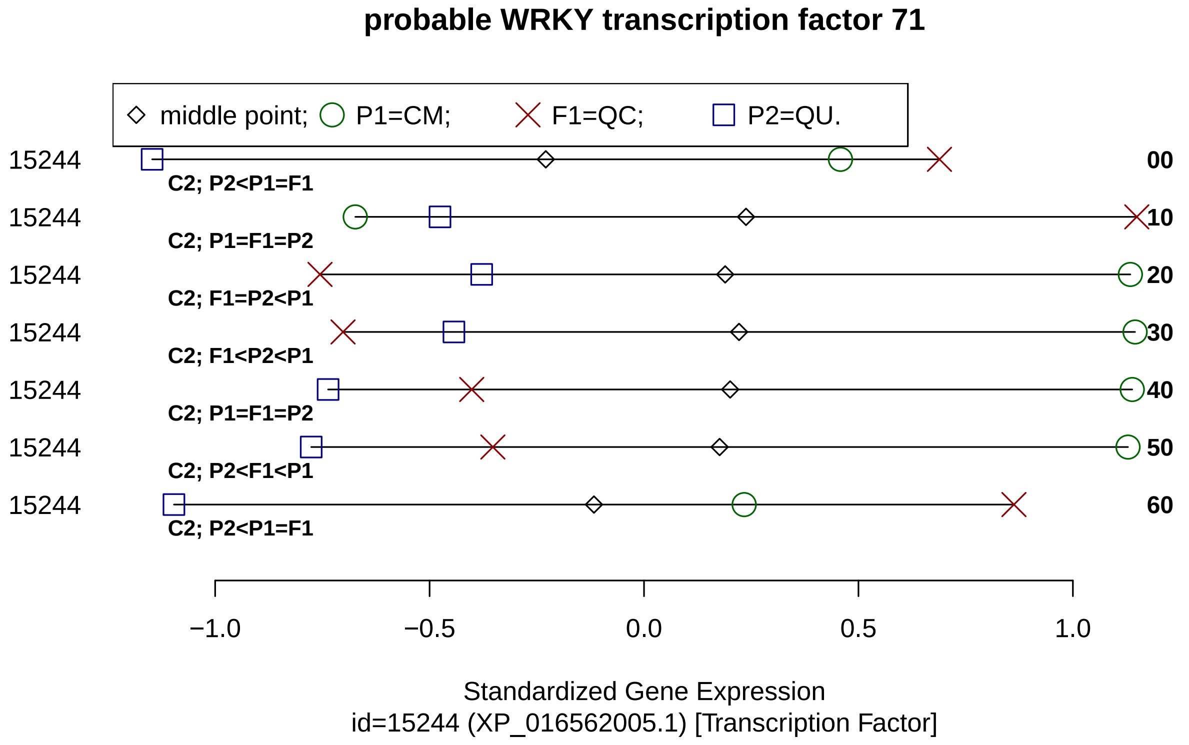

FIGURE 12. Figure resulting from the command "`cross.plot(x=temp.C2, cross='C2')`" (see box of R commands above).

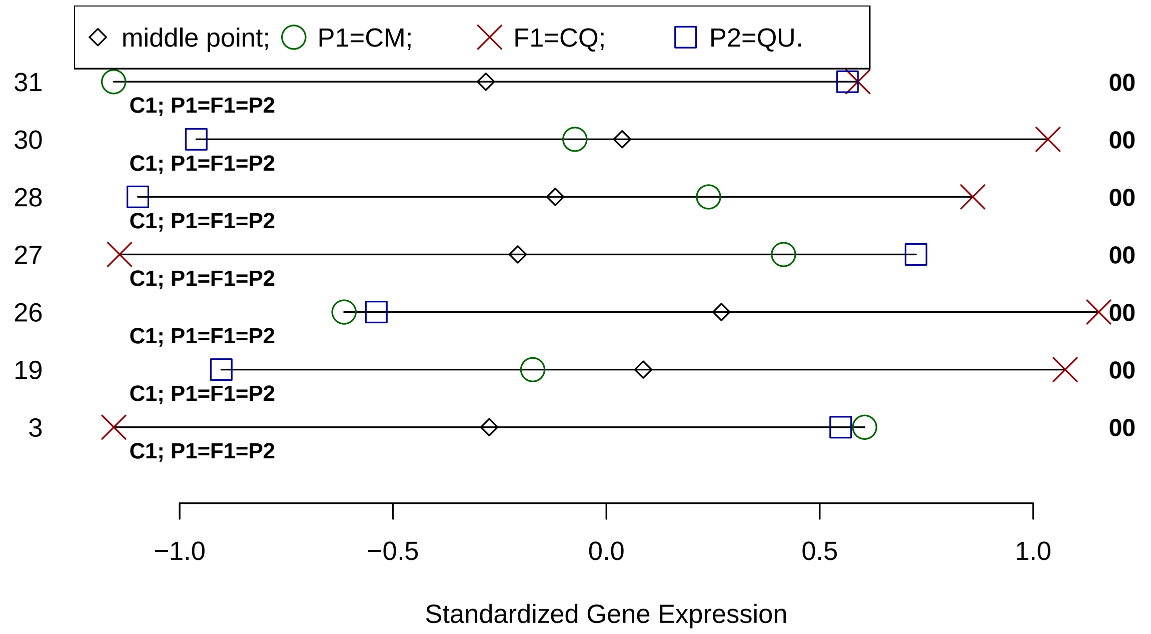

FIGURE 13. Figure resulting from the command "`cross.plot(x=temp.C1.2, cross='C1')`" (see box of R commands above).

References

[1] Michael Ashburner, Catherine A Ball, Judith A Blake, David Botstein, Heather Butler, J Michael Cherry, Allan P Davis, Kara Dolinski, Selina S Dwight, Janan T Eppig, et al. Gene ontology: tool for the unification of biology. *Nature Genetics*, 25(1):25–29, 2000.

[2] Yoav Benjamini and Yosef Hochberg. Controlling the false discovery rate: a practical and powerful approach to multiple testing. *Journal of the royal statistical society. Series B (Methodological)*, pages 289–300, 1995.

[3] Ron Edgar, Michael Domrachev, and Alex E Lash. Gene Expression Omnibus: NCBI gene expression and hybridization array data repository. *Nucleic acids research*, 30(1):207–210, 2002.

[4] Christian Escoto-Sandoval, Alan Flores-Díaz, M Humberto Reyes-Valdés, Neftalí Ochoa-Alejo, and Octavio Martínez. A method to analyze time expression profiles demonstrated in a database of chili pepper fruit development. *Scientific Reports*, 11(1):13181, 2021.

[5] Christian Escoto-Sandoval and Octavio Martínez. *ChiliCross: an R package with data of two crosses of chili pepper (Capsicum annuum L.) during fruit development*, July 2021.

[6] Raivo Kolde. *pheatmap: Pretty Heatmaps*, 2019. R package version 1.0.12.

[7] Octavio Martínez and Christian Escoto-Sandoval. *Salsa: An R package of data mining facilities for* Capsicum *gene expression profiles*, May 2021.

[8] R Core Team. *R: A language and environment for statistical computing.* R Foundation for Statistical Computing, Vienna, Austria, 2013.

[9] Seung Yon Rhee, Valerie Wood, Kara Dolinski, and Sorin Draghici. Use and misuse of the gene ontology annotations. *Nature Reviews Genetics*, 9(7):509, 2008.

[10] Mark D Robinson, Davis J McCarthy, and Gordon K Smyth. edgeR: a bioconductor package for differential expression analysis of digital gene expression data. *Bioinformatics*, 26(1):139–140, 2010.

[11] Barnet Woolf. The log likelihood ratio test (the G-test). *Annals of human genetics*, 21(4):397–409, 1957.
